# Quantifying surface groups on aminated silica nanoparticles of different size, surface chemistry, and porosity with solution NMR, XPS, optical assays, and potentiometric titration

**DOI:** 10.1039/d5na00794a

**Published:** 2025-09-10

**Authors:** Isabella Tavernaro, Isabelle Rajotte, Marie-Pier Thibeault, Philipp C. Sander, Oltion Kodra, Gregory Lopinski, Jörg Radnik, Linda J. Johnston, Andreas Brinkmann, Ute Resch-Genger

**Affiliations:** a Division Biophotonics, Federal Institute for Materials Research and Testing (BAM) Richard-Willstätter-Str. 11 12489 Berlin Germany isabella.tavernaro@bam.de ute.resch@bam.de; b Metrology Research Centre, National Research Council Canada Ottawa Ontario K1A 0R6 Canada andreas.brinkmann@nrc-cnrc.gc.ca; c Clean Energy Innovation Research Centre, National Research Council Canada Ottawa Ontario K1A 0R6 Canada; d Division Surface and Thin Film Analysis, Federal Institute for Materials Research and Testing (BAM) Unter den Eichen 44-46 12203 Berlin Germany

## Abstract

We assessed the quantification of surface amino functional groups (FGs) for a large set of commercial and custom-made aminated silica nanoparticles (SiO_2_ NPs) with sizes of 20–100 nm, prepared with different sol–gel routes, different amounts of surface amino FGs, and different porosity with four methods providing different, yet connected measurands in a bilateral study of two laboratories, BAM and NRC, with the overall aim to develop standardizable measurements for surface FG quantification. Special emphasis was dedicated to traceable quantitative magnetic resonance spectroscopy (qNMR) performed with dissolved SiO_2_ NPs. For the cost efficient and automatable screening of the amount of surface amino FGs done in a first step of this study, the optical fluorescamine assay and a potentiometric titration method were utilized by one partner, *i.e.*, BAM, yielding the amount of primary amino FGs accessible for the reaction with a dye precursor and the total amount of (de)protonatable FGs. These measurements, which give estimates of the minimum and maximum number of surface amino FGs, laid the basis for quantifying the amount of amino silane molecules with chemo-selective qNMR with stepwise fine-tuned workflows, involving centrifugation, drying, weighting, dissolution, measurement, and data evaluation steps jointly performed by BAM and NRC. Data comparability and relative standard deviations (RSDs) obtained by both labs were used as quality measures for method optimization and as prerequisites to identify method-inherent limitations to be later considered for standardized measurement protocols. Additionally, the nitrogen (N) to silicon (Si) ratio in the near-surface region of the SiO_2_ NPs was determined by both labs using X-ray photoelectron spectroscopy (XPS), a well established surface sensitive analytical method increasingly utilized for microparticles and nano-objects which is currently also in the focus of international standardization activities. Overall, our results underline the importance of multi-method characterization studies for quantifying FGs on NMs involving at least two expert laboratories for effectively identifying sources of uncertainty, validating analytical methods, and deriving NM structure–property relationships.

## Abstract 2

BAM and NRC quantified surface amino functional groups (FGs) on 20–100 nm commercial and custom-made silica nanoparticles (SiO_2_ NPs), made with different sol–gel routes, amounts of surface amino FGs, and porosity in a bilateral multi-method study.

## Graphical abstract

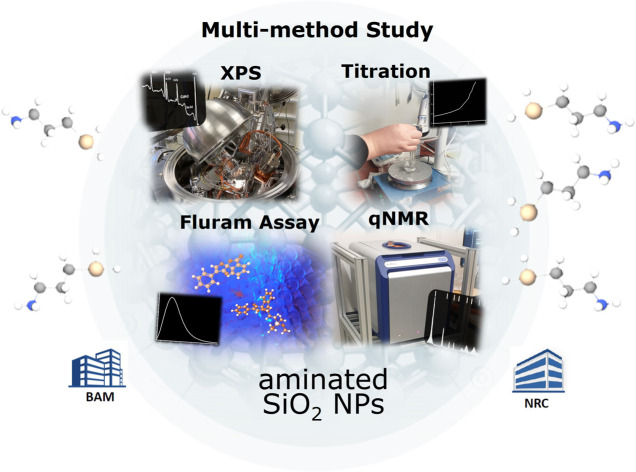

## Introduction

Surface-modified engineered organic and inorganic nanomaterials (NMs) are increasingly used in the life and materials sciences.^1^ Areas of application include medical diagnostics, bioimaging, nano-medicine, sensing, solid state lighting, barcoding, and multiplexing as well as food and consumer products.^2–4^ NM performance depends on key factors such as primary particle size and shape, size and shape distribution, crystallinity, morphology, chemical composition, and surface chemistry, *i.e.*, surface coatings, functional groups (FGs), and ligands.^5,6^ The latter controls NM colloidal stability, surface charge, dispersibility, processibility, and the potential safety of such NMs, when released to the environment. The well-recognized importance of surface chemistry triggered the strong interest of the nanosafety community, standardization organizations, regulators, and NM producers in validated methods for quantifying surface FGs.^7,8^ This includes simple, fast, and cost-efficient methods employing conventional lab equipment that are commonly applied for quality control of NM production processes and NM screening, *e.g.*, in stability studies prior to toxicity and exposure studies, as well as more advanced and expensive techniques providing quantitative and even absolute numbers and traceability. Numerous methods have been reported for identifying and quantifying surface FGs,^5,9–13^ that, however, significantly vary in terms of the information provided, the specific measurand, surface sensitivity, and robustness. Additionally, some of these methods also require a reporter molecule that interacts with the surface FGs to generate a measurable signal.^5^ Other factors relevant for method choice and data interpretation include material-specific constraints and method-specific requirements on sample amount, sample preparation, and stability as well as measurement time, operator skills, and instrument costs.

An emerging method for surface FG quantification on different NMs is solution quantitative nuclear resonance spectroscopy (qNMR), that provides structural and quantitative information on the amount of surface ligands and coatings with a high chemical selectivity; also qNMR is traceable to the SI units mole and kg.^14,15^ Although advanced NMR techniques are increasingly employed to characterize organic ligand shells on dispersed semiconductor quantum dots and gold NPs,^16,18^ and solid state NMR has been used before, *e.g.*, for polymer and silica NMs,^14,19–23^ the potential of broadly available conventional solution NMR for surface analysis of other NMs is still underexplored. Examples are the quantification of surface amino FGs on non-porous and (meso-)porous silica nanoparticles (SiO_2_ NPs), dissolved under strong alkaline conditions, and various FGs and coatings removed from metal oxide NPs.^14,15,19,20,24–29^ Thereby, the amount of the surface ligands and coatings released in solution is measured. The applicability of solution (q)NMR for NM surface characterization and achievable relative standard deviations (RSDs) were recently explored in a first bilateral comparison of NRC and BAM using best practices and in-house protocols by each laboratory, focusing on quantifying surface FGs on a small set of commercial non-porous aminated SiO_2_ NPs with sizes of 20–100 nm, produced by a single manufacturer using the same preparation method, *i.e.*, the common Stöber synthesis and surface functionalization with an amino silane, all in a batch reactor.^27^ These NMs were chosen as silica particles are one of the most abundant and broadly applied engineered NMs, utilized, *e.g.*, as filling materials, food additives, and drug carriers, with the annual production of silica NMs meanwhile amounting to hundreds of thousands of tons.^30,31^

In the present expanded and more advanced bilateral study of BAM and NRC, we aimed (i) to develop reliable, broadly applicable, and eventually standardizable protocols for solution qNMR to quantify surface FGs on large sets of aminated SiO_2_ NPs of varying size, surface morphology, and porosity prepared by different sol gel routes, (ii) to highlight the potential of automatable optical assays and electrochemical titration methods for surface FG screening and their limitations, and (iii) to derive correlations between different analytical methods used for surface FG screening and quantification, thereby underlining the importance and advantages of multi-method characterization concepts. Therefore, large sets of typical commercial aminated SiO_2_ NPs from different manufacturers were assessed, produced by common preparation methods such as the Stöber and the reverse microemulsion approaches.^24–34^ Additionally, sets of non-porous aminated SiO_2_ NPs of different size with varying FG densities were prepared by BAM, utilizing two different sol–gel routes. Prior to the qNMR studies, particle surface FG screening was done by BAM with two simple, cost-efficient, and automatable optical and electrochemical methods. Such methods are applied by many NM producers for the quality control of their production processes to a broad variety of NPs of different chemical composition.^5,20^ As conductometry is of limited use for metal oxide NPs as explored here, we focused on a potentiometric back titration in the present study. The resulting amount of primary amino FGs accessible for the reaction with the dye precursor and the total amount of (de)protonatable FGs measured by the colorimetric and fluorometric fluorescamine (Fluram) assay and the electrochemical pH titration were then used to estimate the minimum and maximum number of surface amino FGs on the different aminated SiO_2_ particles and to obtain information on particle surface morphology. To explore and fine-tune the workflow of the qNMR measurements, which chemo-selectively provide the total amount of surface ligand molecules released by NM dissolution, with the goal to identify and minimize sources of uncertainties arising from sample preparation, data acquisition, and data evaluation steps, NRC and BAM performed a bilateral study on qNMR. This provided the basis to utilize data comparability and relative standard deviations (RSDs) of both labs as quality measures for method optimization. This qNMR study was complemented by a bilateral X-ray photoelectron spectroscopy (XPS) study of selected samples, yielding the near surface nitrogen (N) to silicon (Si) ratio of the solid aminated SiO_2_ NPs deposited onto a solid substrate. Thereby, an established method for surface analysis, was included in this comparison, which is currently also in the focus of international standardization. Subsequently, the potentiometry, qNMR, and XPS data were correlated, to validate the former screening method and as a first step to traceable XPS measurements. Overall, our results yield fine-tuned workflows for quantifying amino silanes on dissolved aminated SiO_2_ NPs and highlight the advantages of multi-method characterization schemes which combine information from methods that rely on different mechanisms of signal generation, require different sample preparation steps, and target different, yet commonly correlated measurands. This approach enables an efficient method cross-validation, paves the road to reliable, comparable, and eventually standardized measurements of surface FGs, and increases our understanding of NM structure–property relationships.

## Experimental

### Materials

All chemicals, reagents, and solvents were of analytical grade or higher and used as received, unless otherwise stated, while all aqueous solutions and buffers were prepared with ultrapure water (MilliQ-water, 0.055 μS m^−1^; Merck Milli-Q® IQ 700 device). Commercial non-porous aminated SiO_2_ NPs were purchased from NanoComposix (NC, USA) as ethanolic suspensions (≈10 mg mL^−1^), referred to in the following as “NC-size” samples. These NPs were synthesized by the Stöber process and functionalized with (3-aminopropyl)triethoxysilane (APTES) in a post-synthetic grafting step. Mesoporous 100 nm NPs from NC were also studied. 50 nm sized amorphous, aminated SiO_2_ NPs synthesized by a reverse microemulsion approach were obtained from HiQ-Nano (Italy, HiQ-50), while another 50 nm sized batch synthesized by the Stöber process was obtained from microparticles GmbH (Germany, MP-50).

#### Nanoparticle syntheses and surface modification (BAM SiO_2_ NPs NH_2_)

Amorphous, non-porous SiO_2_ NPs with sizes of 25 nm, 50 nm, and 100 nm were synthesized at BAM by two sol–gel routes as described in previous work,^35^ using tetraethoxysilane (TEOS, Sigma-Aldrich, Germany) as the silica precursor and ammonia (abcr GmbH, Germany) or l-arginine (Carl Roth GmbH, Germany) as alkaline catalyst (SI Sections 1.1 and 1.2). The surface of the SiO_2_ NPs was modified in a postsynthetic grafting step with APTES in ethanol (SI Section 1.3).^36^

#### Nanoparticle characterization

Particle size and surface charge were characterized at BAM by dynamic light scattering (DLS SI Section 2.2) and zeta potential measurements using a Malvern Panalytical Zetasizer Nano ZS equipped with a 633 nm laser (SI Section 2.1). The morphology of the custom-made and commercial SiO_2_ NPs was obtained from transmission electron microscopy (TEM) micrographs recorded with a Tecnai G2 20 S-Twin microscope (FEI Company, USA), SI Section 2.4. Aqueous dispersions of the larger 80 nm and 100 nm NPs were also characterized by nanoparticle tracking analysis (NTA) using the NanoSight LM 10 system (Malvern Panalytical) equipped with a 405 nm laser (SI Section 2.3) to validate the number-based hydrodynamic diameter (*d*_h,0_) and the gravimetrically derived particle number concentrations (PNC) using NP diameters from TEM. The mass fraction of SiO_2_ NPs in one vial of each batch was gravimetrically determined in triplicate by drying 0.25 mL of the NP dispersion in plastic centrifuge tubes (2 mL safe lock Eppendorf tubes, Eppendorf GmbH, Germany) overnight at 100 °C. The specific surface area of the custom-made SiO_2_ NPs was estimated from TEM diameters, using typical silica densities of 2.09 g cm^−3^ and 2.20 g cm^−3^ for the particles obtained by the l-arginine and the Stöber method (SI Section 2.5).^37^ An overview of the characterization results is given in Table S1.

#### Quantification of amino FGs with an optical assay and potentiometric back titration

The reporter-accessible number of surface amino FGs of the custom-made (BAM SiO_2_ NH_2_) and commercial aminated SiO_2_ NPs was screened by BAM with a semi-automated optical assay using the dye precursor 4′-phenylspiro[2-benzofuran-3,2′-furan]-1,3′-dione (Fluram®, fluorescamine).^36^ For selected samples, the number of (de)protonatable FGs was also determined with a fast and inexpensive potentiometric back titration approach using protons as ultrasmall reporters for FG screening (SI Section 4).^36^

#### Quantitative nuclear resonance spectroscopy (qNMR)

The total amount of amino FGs was determined by qNMR, first employing protocols adapted from our previous work,^27^ which were then stepwise optimized (SI Section 5). Therefore, the aminated SiO_2_ NPs were removed from ethanolic dispersion by centrifugation (BAM: centrifuge Eppendorf 5424 RG: speed of 21 000 rcf; NRC: centrifuge Fisher Scientific AccuSpin Micro 17R, speed of 17 000 rcf), dried in centrifuge tubes (safe lock 1.5 mL or 2.0 mL, Eppendorf GmbH or Fisherbrand Microcentrifuge tubes, 2.0 mL) at an elevated temperature overnight, weighed with an ultra-micro balance (BAM: Cubis MCM 6.7 (Satorius), NRC: XP-6U (Mettler Toledo)), and dissolved by addition of a 1 M sodium deuteroxide solution (NaOD, Sigma-Aldrich) in D_2_O (Sigma-Aldrich) at 50 °C. The tube drying procedure was later standardized, using a temperature of 100 °C. For qNMR, ultrapure maleic acid (TraceCERT®, Sigma-Aldrich) was added as an internal standard (2H, 6.3 ppm) to the sample solutions. Maleic acid does not display signals in the frequency window used for amino FG quantification at 2.4 ppm and 0.3 ppm originating from the two aliphatic CH_2_ groups of the 3-aminopropyl groups grafted to the SiO_2_ NP surface. The signal at 1.3 ppm was not utilized for FG quantification due to its proximity to the methyl signal of residual ethanol. At BAM the NMR experiments were performed on a 600 MHz JEOL ECZ spectrometer, where a 90° pulse angle, a pulse delay of 50 s, 64 scans, an acquisition time of 3.6 s, and a spectral width of 30 ppm were used. At NRC, a Bruker 400 MHz Avance III spectrometer was used together with a 90° pulse length of around 17 μs, a pulse delay of 50 s, 32 scans, and acquisition times of 8.3 s and 5.5 s together with spectral widths of 20 ppm and 30 ppm, respectively.

#### X-ray photoelectron spectroscopy (XPS)

Similar procedures were used to pretreat and prepare samples for XPS analysis at both BAM and NRC (SI Section 6). Thin films were prepared by drop-casting onto ozone cleaned Au-coated substrates as described elsewhere.^38^ XPS measurements were performed at BAM with a Quantes photoelectron spectrometer manufactured by Ulvac-PHI (Chanhassen, MN, USA) and measurements at NRC with an Axis Ultra DLD spectrometer (Kratos Analytical, Manchester, UK). The spectrometer used at BAM uses a micro-focused X-ray beam which facilitates improved spatial resolution but may increase the probability of beam damage as discussed in the results section.

## Results and discussion

**Fig. 1:**
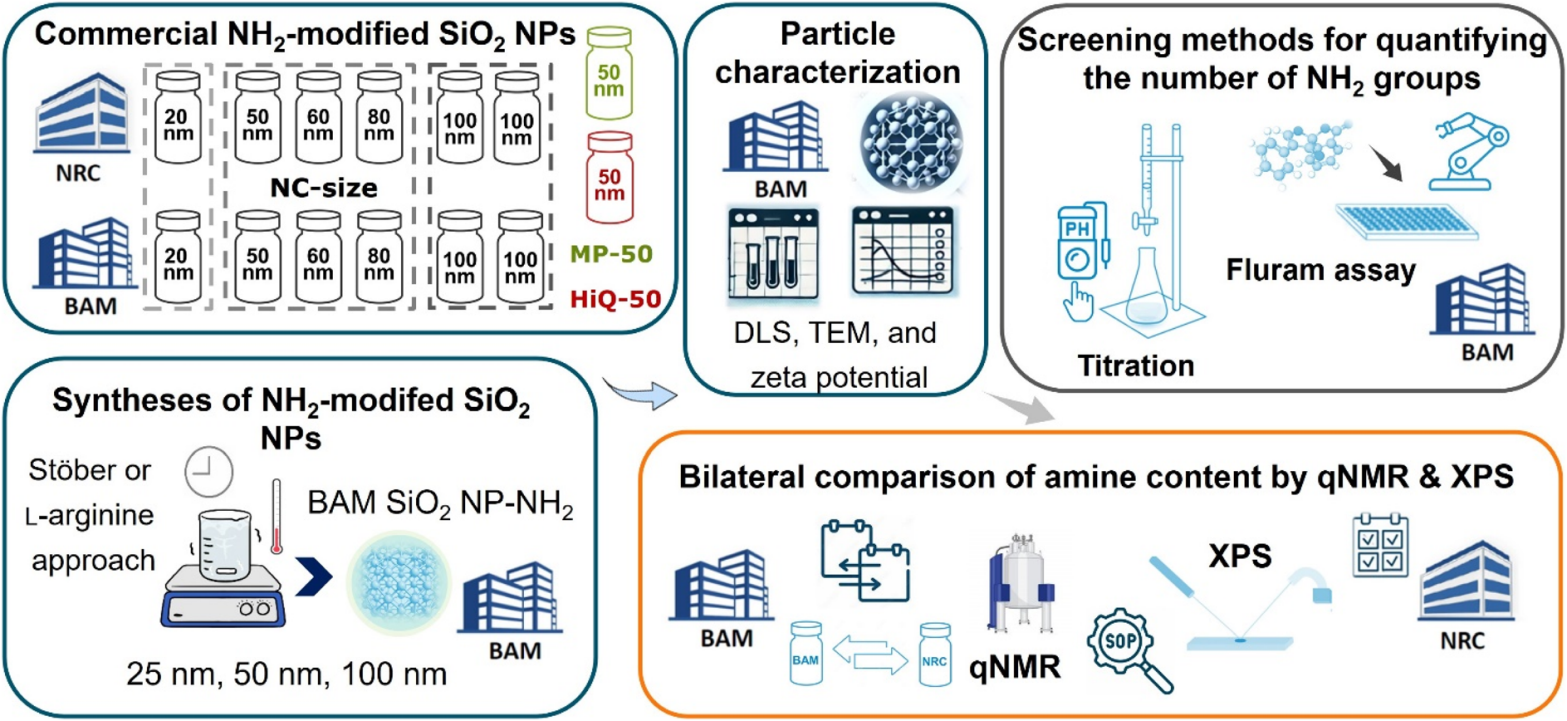
Overview of the multi-method characterization study, summarizing the aminated SiO_2_ NP samples obtained from nanocomposix (NC), HiQ-nano, (HiQ) and microparticles GmbH (MP), as well as those synthesized at BAM *via* the Stöber and l-arginine sol–gel routes.^17^ The analytical methods employed for particle characterization prior to FG quantification included structural analysis using dynamic light scattering (DLS), zeta potential measurements, transmission electron microscopy (TEM), and nanoparticle tracking analysis (NTA), see SI for more details. Rapid screening and quantification of the surface amino FGs were performed at BAM using an automated fluorescamine (Fluram) assay and a potentiometric back titration method, providing values for reporter accessible FGs and total (de)protonatable FGs, respectively. A bilateral comparison between BAM and NRC was conducted using traceable and chemo-selective solution-state quantitative NMR (qNMR) to measure the total amount of amino silane molecules, measured with a refined qNMR workflow. X-ray photoelectron spectroscopy (XPS) was also employed to determine the N/Si ratio in the near-surface region of the particles with an information depth of about 5 nm.

The production and quality control of engineered NMs, as well as stability monitoring and reliable, comparable NM risk assessment studies, all require validated methods. This includes standardized protocols for sample preparation, measurement, and data evaluation for NM surface characterization, with well-known measurement uncertainties. Aiming to develop standardized methods for surface FG analysis with a special focus on traceable and chemo-selective solution qNMR, the metrology institutes NRC and BAM conducted a multi-method characterization study of a large set of representative commercial and custom-made aminated SiO_2_ NPs, with sizes of 20 to 100 nm, obtained by different commonly utilized preparation methods with varying amount of amino FGs and varying porosity. Thereby different, yet most likely correlated measurands and optical and electrochemical screening methods utilized by NM manufacturers were explored. The commercial aminated SiO_2_ NPs were synthesized with the Stöber and reverse microemulsion methods, while BAM prepared sets of non-porous aminated SiO_2_ NPs by the Stöber and the less frequently used l-arginine approach.^39–42^ These synthesis methods and the amount of amino silane utilized for surface functionalization can result in different surface morphologies and mono- or multilayer surface structures of the amino silanes.^41–43^ Particle surface FG screening prior to the qNMR studies was done by BAM with the fluorescamine (so-called Fluram) assay and a potentiometric back titration method, yielding the number of amino FGs, that react either with the precursor dye 4′-phenylspiro[2-benzofuran-3,2′-furan]-1,3′-dione,^22,36^ or with ultrasmall protons.^5,9,36^ The latter presents the total amount of FGs accessible for protonation. The dye-reporter-accessible and the total protonatable FG content obtained by these two cost-efficient and automatable screening methods can also provide information on the SiO_2_ NP surface structure. This can be relevant for interpreting qNMR and XPS measurements. By contrast, chemo-selective qNMR directly measures the total amount of amino FGs by utilizing the NMR signals of the amino silane molecules released from known amounts of previously dried aminated SiO_2_ NPs after dissolution under alkaline conditions.^15,19,27^ As RSDs and data comparability of the qNMR workflows can be affected by uncertainties originating from sample preparation, measurement, and data evaluation steps, these steps were jointly examined in this study (SI, Section 5, qNMR spectroscopy) by NRC and BAM. The qNMR study was complemented by XPS measurements of selected aminated SiO_2_ NPs deposited on a solid support. These measurements provide the atomic composition of the aminated SiO_2_ NPs in the near surface region, with an effective probing depth of about 5 nm.^19,44^Fig. 1 shows an overview of the workflows utilized for this multi-method study, including samples and characterization methods used by BAM to determine the intrinsic parameters of shape, particle size (number-based size distribution: DLS and NTA (*d*_h,0_); TEM), and particle number concentrations (PNC), see SI Section 2 for details, and the methods employed for quantifying the number of amino FGs, assessing different measurands. To demonstrate the influence of the method-specific measurands and method-inherent limitations on FG determination which are frequently underestimated, we correlated the data obtained with these different methods. Our results, which highlight the wealth of information that can be gained from multi-method studies of the same nano-objects, will pave the road to more multi-method studies of systematically chosen sets of the same nano-objects in the future, as required for a better understanding of the applicability of surface analysis methods.

### Characterization of the physicochemical NP properties

All custom-synthesized NP samples constituted of non-porous, positively charged, colloidally stable SiO_2_ NPs with zeta potentials ranging from 11.0 to 47.2 at physiological pH values, that increased with the APTES amount employed for particle surface grafting (see SI, Section 2, Particle characterization). The results of the TEM and DLS measurements of the commercial NPs agree well with the data sheets provided by the manufacturers. For comparison to the amine content assessed by the various methods (SI, Table S1), the amount of amino FGs was calculated for each particle size for an amine monolayer, assuming 4 amine molecules per nm^2^ (ref. 45) and using the specific surface areas obtained from TEM particle diameters (SI Section 2.5). This gave amino FG amounts per monolayer of about 730 μmol g^−1^ (NC-20) to 169 μmol g^−1^ (NC-100 non-porous) and 4178 μmol g^−1^ (NC-100 mesoporous) for the commercial particles, as well as 995 μmol g^−1^ (BAM SiO_2_-25 NH_2_) (Stöber) to 189 μmol g^−1^ (BAM SiO_2_-100 NH_2_) for the custom-made particles. The two 50 nm sized commercially available particles from HiQ-nano and microparticles GmbH have estimated, calculated monolayer coverages of amino FGs of 329 μmol g^−1^ and 283 μmol g^−1^, respectively.

### Fast screening of surface amino FGs with the optical Fluram assay and a potentiometric back titration

First, the amount of surface amino FGs on selected 50 nm and 100 nm aminated SiO_2_ NPs, prepared by different synthesis methods by commercial producers and by BAM, was assessed using the optical Fluram assay and a potentiometric back titration at BAM. The optical assay, recently automated by us, is ideal for the rapid screening of surface amino FGs that are accessible to the assay's dye reporter molecule, requiring only minimal particle consumption. This measurement provides a lower limit for the amount of surface amino FGs due to the relatively large size of the dye reporter compared to, *e.g.*, protons. It is particularly relevant for applications involving further labeling steps, such as the covalent attachment of dyes, PEG ligands, biomolecules, or other target-specific recognition moieties. Therefore, such optical assays, which can be performed using widely available and cost-efficient instrumentation, are often employed by particle producers.^22,36^ The potentiometric back titration,^36,46^ which utilizes ultrasmall protons as signal-generating reporters, is also a robust, fast, and cost-efficient method. However, it consumes more material and lacks chemo-selectivity as every protonatable FG is measured. This can result in an overestimation of the amount of surface amino FGs, *e.g.*, for SiO_2_ NPs modified with surfactants or several ligand types, as well as non-purified samples which contain an excess of non-surface bound amino silanes.

**Fig. 2:**
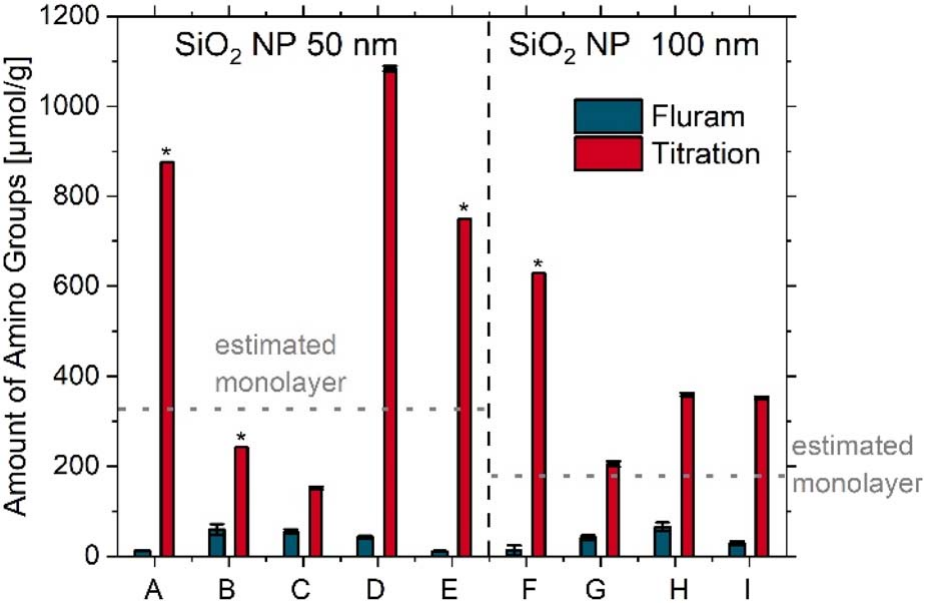
Screening of the amount of surface amino FGs for 50 nm sized SiO_2_ NPs prepared by different synthesis methods, *i.e.*, the Stöber, the microemulsion, and the l-arginine approach (left) and 100 nm sized SiO_2_ NPs varying in porosity and/or amount of APTES used for surface grafting (right): A = HiQ-50; B = MP-50; C = NC-50; D = BAM SiO_2_-50 NH_2_ high (arginine); E = BAM SiO_2_-50 NH_2_ low (arginine); F = NC-100 (mesoporous); G = NC-100 (non-porous); H = BAM SiO_2_-100 NH_2_ high; I = BAM SiO_2_-100 NH_2_ low. * = single experiment.

As shown in Fig. 2, not only the particle size and amount of amino silane molecules applied for post-synthetic grafting but also the preparation method and particle morphology can influence the reporter accessible and total amount of surface amino FGs. While the amorphous, non-porous SiO_2_ NPs from NC used in this study typically carry at most one monolayer of surface amino FGs,^11^ the APTES concentration used for grafting the non-porous SiO_2_ NPs from BAM was varied. As a result, the amount of surface FGs is likely to exceed a monolayer in most samples. This situation can occur not only in research samples but also in commercial SiO_2_ NPs, which are often synthesized using different methods and with varying amounts of surface amino FGs.^19,47^

This is shown by the results obtained for HiQ-50, synthesized *via* the reverse microemulsion method and exhibiting a high amount of amino FGs, and MP-50, synthesized with the Stöber approach, which shows a higher amount of amino FGs than the NC-50 sample. In all cases, the ratio of the total and accessible amount of amino FGs is higher for samples with multilayers. The three samples (B, C, G) with approx. 1 monolayer or less display a smaller difference between the amount of total and accessible amino groups. This can have, *e.g.*, important consequences for further functionalization steps. These results highlight that the amount of amino silane applied for surface grafting should be chosen according to the desired application, *e.g.*, focusing on a very good monodispersity, surface charge or a larger amount of (bio-)conjugatable amino FGs. In addition to the non-porous aminated SiO_2_ NPs, 100 nm aminated mesoporous SiO_2_ NPs with a larger surface area from NC were measured. Thereby, possible sample-related effects were exemplarily explored as well as the response of the different characterization methods to these effects. This follows from a comparison of non-porous and mesoporous samples. Non-porous NC-100 and the two BAM SiO_2_-100 NH_2_ samples display amino FG amounts slightly exceeding the values estimated for a dye reporter-labelled APTES monolayer (SI, Section 3 on screening of the accessible number of amino groups *via* an optical assay) while the mesoporous sample revealed only 2% of the Fluram molecules calculated for a monolayer. This suggests that although the Fluram molecules with a size of about 0.7 nm^2^ can enter the 3 nm pores, immobilization of one reporter molecule near the pore entrance can block a pore, thereby preventing the coupling of other Fluram molecules to free amino FGs in this pore. These results demonstrate the importance of quantifying the amount of amino FG with methods focussing on different, yet somehow correlated measurands for an accurate determination of surface FGs intended for different applications. While the total amount of amino FGs is almost the same for the potentiometric back titration, according to the Fluram assay, samples BAM SiO_2_-100 NH_2_ low and high vary in accessible amino FG amount by a factor of 2.25. This result agrees well with the differences in zeta potential measurements for these two samples. This points to different surface chemistries of these samples, *i.e.*, differently accessible, or sterically differently hindered surface amino FG groups, most likely indicating a different organization of the amino FGs in the surface multilayers.

### Assessing qNMR workflows for quantifying surface amino FGs

**Fig. 3:**
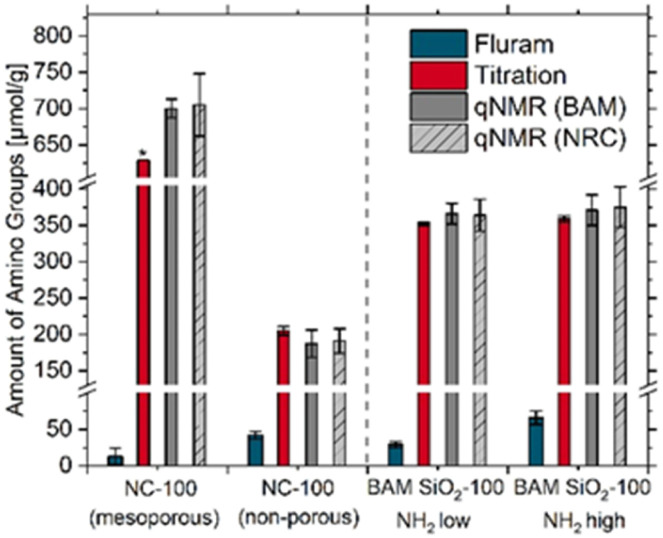
Comparison of the results obtained with the optical Fluram assay (blue bars) and the potentiometric titration (red bars) used for surface amino FG screening and the bilateral comparison of the qNMR measurements (grey bars) of the different 100 nm sized SiO_2_ NPs. NP screening was performed at BAM with the samples provided to BAM + NC-100 (mesoporous), while the qNMR measurements were carried out at BAM and NRC with the different bottles provided to the institutes, respectively. The individual values are provided in Table 1; * = single experiment.

Next, BAM and NRC performed a bilateral comparison of qNMR measurements for the quantification of surface amino FGs on different sets of aminated SiO_2_ NPs with the aim of improving data comparability and reducing RSD values compared to the previous study.^27^ qNMR measurements chemo-selectively provide the total amine content in the samples after dissolving the SiO_2_ NPs, that is not limited by the FG accessibility to the reaction with a reporter and reporter size.^15,47^ Previously, such qNMR measurements were solely conducted with a small set of amorphous, non-porous aminated SiO_2_ NPs from one company NC. First, different types of 100 nm sized aminated SiO_2_ NPs from NC and BAM were assessed (Fig. 3). This size was chosen as our previous solution qNMR measurements demonstrated a good data comparability for commercial SiO_2_ NPs of this size from NC.^27^ We initially optimized the qNMR method, developing a single protocol for use in both labs. First, we adopted an approach that involved the pelleting an aliquot of NP suspension by centrifugation, removal of the supernatant followed by drying the sample and weighing the silica material prior to the hydrolysis procedure. This ensures that any free amino silane released from the surface during storage is removed in the supernatant and not included in the total amine content of the sample. In this step, it is important to dry the tube used in an oven at approximately 100 °C prior to the experiment as the mass loss due to drying can be as large as 1 mg, close to 10% of the silica mass used. Second, the amount of sample to be used per hydrolysis was set to ∼12 mg; this was tested by experiments using 10 mg *vs.* 20 mg of silica per hydrolysis experiment. This indicated that the amine content was the same within the standard deviation of the measurement. Third, the qNMR data acquisition protocol was standardized to ensure a similar number of data acquisition scans and the use of the same relaxation time (*T*_1_) for both labs. Finally, the data evaluation was also standardized, ensuring that satellite peaks were included in the integration and verifying that data analysis of the same data sets by two different operators gave results that were the same within the standard deviation of the measurement for several samples. As to be expected, main sources of uncertainty are the weighing, the NP dissolution, and data evaluation steps. Full details of the optimization experiments and the finally optimized protocol are summarized in the SI, Section 5.

The potentiometric back titration measurements, which determine the total amount of protonatable amino FGs, were then compared with the qNMR results, giving the total amount of amino FGs.

The potentiometric titration yielded surface amino FG densities of 629 μmol g^−1^ (15% of the estimated monolayer) and 205 μmol g^−1^ (121% of the estimated monolayer) for the mesoporous and non-porous NC-100 samples and values of 352 μmol g^−1^ and 359 μmol g^−1^ (∼180% of the estimated monolayer) for the two BAM SiO_2_-100 NH_2_ samples.

These data are comparable with the results of the qNMR measurements. This highlights the excellent agreement of the results of the potentiometric back titration and the qNMR measurements for the non-porous silica NPs, despite the reduced sensitivity and selectivity of the electrochemical method. For the non-porous NC-100 sample, both labs obtained an amino FG amount of about 195 μmol g^−1^, while for the 100 nm sized BAM SiO_2_ NPs prepared with low and high APTES concentrations, the results of the qNMR measurements with the previously centrifuged, dried, and then dissolved SiO_2_ NPs match within the RSDs of the qNMR measurements. The BAM qNMR measurements yielded values of 366 ± 14 μmol g^−1^ and 371 ± 21 μmol g^−1^, respectively, in excellent agreement with the NRC data. This is in contrast with the findings of the Fluram assay underlining the different accessibility of the surface amino FGs introduced by APTES grafting for dye labeling. For the mesoporous sample, both qNMR measurements showed less agreement with the titration method, which yielded a lower amount of amino FGs. However, the qNMR results obtained for the mesoporous sample NC-100 excellently matched, with values of 700 ± 13 μmol g^−1^ and 705 ± 43 μmol g^−1^ of BAM and NRC, respectively. This indicates a much higher amino FG content compared to the non-porous samples, which is ascribed to the high internal surface area. This result is attributed to a slight hindrance of the mobility and diffusion of the proton reporters by the mesoporous silica matrix. The repeatability of the qNMR data of each lab is good with typical RSDs <6%. A paired *t*-test verifies that the differences between the means for the two labs are not significantly different (0.05 level) for all samples, indicating a good reproducibility across the two labs. qNMR measurements performed after exchanging the samples between both labs did not reveal bottle-to-bottle variations between the two labs. This indicates a good transport stability of the samples, as well as a lack of aging-induced changes in amino FG amount after 8 months (SI, Table S3). Overall, our qNMR results do not seem to be especially affected by the different surface morphologies imposed by the mono- or multilayer structures of covalently bound amino silane molecules of the aminated SiO_2_ NP samples representatively studied. Also, qNMR measurements seem to be suited for quantifying amino FGs of mesoporous silica samples. In addition, the similar trends displayed by the data sets obtained by qNMR and the potentiometric back titration support the applicability of the latter method for the screening of surface amino FGs on non-porous silica NPs and most likely also for mesoporous silica.

### Method comparison for differently sized aminated SiO_2_ NPs

**Fig. 4:**
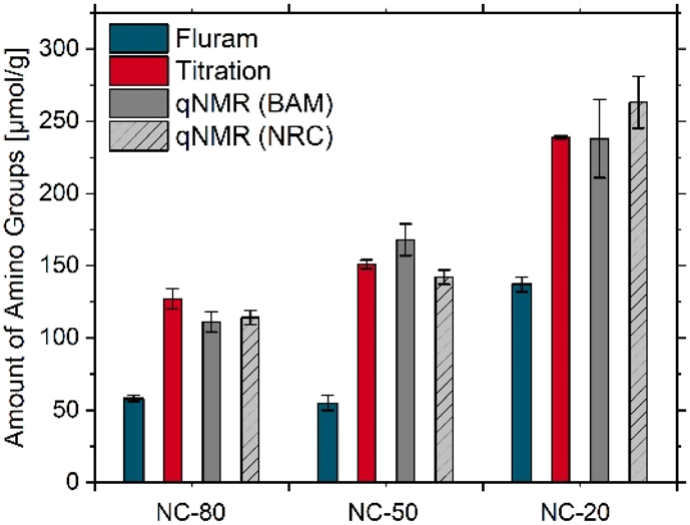
Comparison of the screening results obtained for differently sized commercial non-porous SiO_2_ NPs from NC with the optical Fluram assay (blue bars) and the potentiometric titration (red bars), both carried out by BAM, with the qNMR measurements (grey bars) performed by BAM and NRC. Screening of the NC samples was done by BAM with the SiO_2_ NPs bottles obtained by BAM from NC, while the qNMR measurements were carried out at BAM and NRC with the different SiO_2_ NPs bottles provided by NC to each institute (involving sample exchange between BAM and NRC to assure measurements of identical samples).

**Fig. 5:**
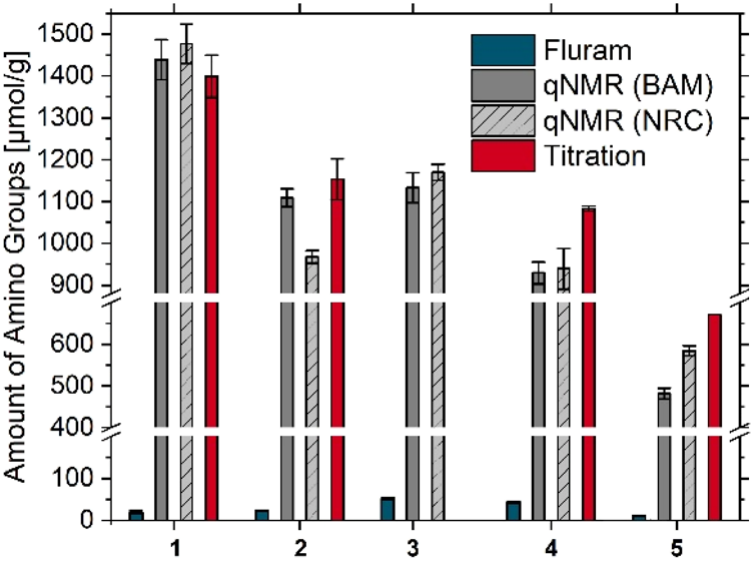
Comparison of the screening results obtained with the optical Fluram assay (blue bars) and the potentiometric titration (red bars) performed at BAM with the results of the bilaterally done qNMR measurements (grey bars) of the differently sized custom-made silica particles with different surface amino FGs and synthesis approaches. The individual values are given in Table 1; 1 = BAM SiO_2_-25 NH_2_ high (Stöber); 2 = BAM SiO_2_-25 NH_2_ high (arginine); 3 = BAM SiO_2_-50 NH_2_ high (Stöber); 4 = BAM SiO_2_-50 NH_2_ high (arginine); 5 = BAM SiO_2_-50 NH_2_ low (arginine).

To study potential influences of SiO_2_ NP particle size and synthesis methods and to further fine-tune our qNMR workflow and protocols, we extended our multi-method characterization approach to smaller amorphous, non-porous silica particles from NC and BAM. A representative overview of the results obtained for 20/25, 50, and 80 nm SiO_2_ NPs from NC and synthesized at BAM using two methods is shown in Fig. 4 and 5. The respective individual values can be found in Table 1. Further results of the joint qNMR measurements with differently sized aminated SiO_2_ NPs from BAM prepared by two different sol–gel routes are provided in the SI in Fig. S15–S18.

**Table 1:** Overview of the obtained amounts of surface amino FGs on aminated SiO_2_ NPs in this study, as determined by two screening methods (optical Fluram assay and potentiometric back titration), and the bilateral comparison (qNMR and XPS measurements) performed at BAM and NRC. n.d. = not determined; * = single experiment; ‡ = total amine content without purification step. A control experiment (see SI, Fig. S15 and S16) showed that 826 ± 123 μmol g^−1^ was on NPs and the remainder in the supernatant. XPS measurements were performed with the different bottles from the same lot provided to each institute. The XPS results of NC-100a present measurements of the original BAM bottle

Particle	Screening		Bilateral comparison			
	Fluram assay [μmol g^−1^]	Potentiometric titration [μmol g^−1^]	qNMR		XPS	
			BAM [μmol g^−1^]	NRC [μmol g^−1^]	BAM N/Si survey	NRC N/Si survey
NC-100 (mesoporous)	13 ± 11	629*	700 ± 13	705 ± 43	n.d.	0.045 ± 0.005
NC-100a (non-porous)	42 ± 5	205 ± 6	187 ± 19	191 ± 17	0.105 ± 0.002	0.126 ± 0.006
NC-100b (non-porous)	40 ± 13	n.d.	199*	202*	0.109 ± 0.006	0.110 ± 0.005
BAM SiO_2_-100 NH_2_ low	29 ± 4	352 ± 3	366 ± 14	364 ± 22	0.122 ± 0.005	0.138 ± 0.003
BAM SiO_2_-100 NH_2_ high	66 ± 9	359 ± 4	371 ± 21	375 ± 28	0.122 ± 0.010	0.138 ± 0.004
NC-80	58 ± 2	127 ± 7	111 ± 7	114 ± 5	0.042 ± 0.003	0.046 ± 0.010
NC-60	57 ± 5	85 ± 1	143 ± 8	154 ± 5	0.040 ± 0.005	0.047 ± 0.003
NC-50	55 ± 5	151 ± 3	168 ± 11	142 ± 5	0.034 ± 0.004	0.045 ± 0.003
BAM SiO_2_-50 NH_2_ low (Stöber)	39 ± 6	n.d.	459 ± 24	968 ± 23	n.d.	n.d.
BAM SiO_2_-50 NH_2_ high (Stöber)	52 ± 2	n.d.	1133 ± 36	1170 ± 19	n.d.	n.d.
BAM SiO_2_-50 NH_2_ low (arginine)	11 ± 1	671*	482 ± 13	584 ± 12	n.d.	n.d.
BAM SiO_2_-50 NH_2_ mid (arginine)	22 ± 2	719*	872 ± 18	640 ± 16	n.d.	n.d.
BAM SiO_2_-50 NH_2_ high (arginine)	43 ± 3	1083 ± 6	929 ± 26	939 ± 48	n.d.	n.d.
NC-20	137 ± 5	239 ± 1	238 ± 27	263 ± 18	0.027 ± 0.003	0.030 ± 0.003
BAM SiO_2_-25 NH_2_ high (Stöber)	20 ± 3	1399 ± 51	1439 ± 48‡	1477 ± 47‡	n.d.	n.d.
BAM SiO_2_-25 NH_2_ low (arginine)	n.d.	n.d.	870 ± 10	715 ± 27	n.d.	n.d.
BAM SiO_2_-25 NH_2_ high (arginine)	22 ± 1	1153 ± 49	1109 ± 21	± 15	n.d.	n.d.

As follows from Fig. 4 and 5, all methods revealed relatively similar trends of the amount of surface amino FGs, although the values vary with the method or measurand as to be expected. As shown in Fig. 4, for the NC samples made by the Stöber method, the qNMR results of both labs agree well with a paired *t*-test indicating that none of the differences in means for the two labs are significantly different (0.05 level). The surface amino FG amount detected with the Fluram assay was always considerably lower than the qNMR results and revealed a size-dependency, with a decrease of the amount of dye reporter-accessible amino FGs from 137 μmol (NC-20) to about 60 μmol for the larger sized NC samples.

The potentiometric back titration (Fig. 4, red bars; SI, Fig. S14) yielded values of 239 μmol g^−1^ (NC-20), 151 μmol g^−1^ (NC-50), and 121 μmol g^−1^ (NC-80) for the total surface amino FGs. These values lay between one-third to one-half of one APTES monolayer for the estimated coverage.

Importantly, the results of the qNMR and electrochemical titration studies revealed a very similar trend, highlighting the good correlation between both measurements despite the different chemo-selectivity. A comparison of the two synthesis routes Stöber and Arginine, utilized by BAM, revealed differences in the total amount of surface amino FGs determined by qNMR (Fig. 5). Even if the particle sizes were similar, for the l-arginine approach, a significantly lower amount of surface amino FGs was obtained compared to the Stöber method. The amino FG amount derived from the potentiometric back titration revealed a similar trend as the qNMR data as observed before. In addition, we did not observe an influence of the operator for the titration experiments (SI, Fig. S14) and the qNMR measurements (SI, Fig. S19).

The trend for aminated SiO_2_ NPs prepared by the Stöber and l-arginine methods displayed in Fig. 5 suggests that according to the results obtained for sample BAM SiO_2_-50 NH_2_ high (arginine), for sample BAM SiO_2_-50 NH_2_ high (Stöber), the potentiometric titration should also provide a value closely matching with the qNMR data. This number should exceed the value measured for BAM SiO_2_-50 NH_2_ high (arginine) as suggested by the analytical data obtained for SiO_2_ NPs prepared by these two sol–gel methods. However, for the BAM-SiO_2_-25/50 NH_2_ (arginine), the potentiometrically obtained amino FG amount exceeded the amount of surface amino FGs measured by qNMR. A possible explanation could be the presence of l-arginine molecules containing a primary amino FG which can be protonated. The measurements with BAM SiO_2_-25 NH_2_ high (Stöber) sample also highlight the limitations of a manually performed potentiometric back titration. In this case, at higher amino FG concentrations, the volume of addition of a single drop can lead to an overtitration and result in an underestimation of the actual values. This can be, however, overcome by automation.

The results in Table 1 and Fig. 5 indicate that four of the samples (50 nm, Stöber low; 25 nm arginine high; 25 nm arginine low; 50 nm arginine mid) have significant differences in mean values for the two labs based on a paired *t*-test. Subsequently, we performed additional experiments to identify possible explanations for the poorer reproducibility of the qNMR data for these samples most of which were prepared with the l-arginine approach. First, the results of the Fluram assay revealed that the amount of accessible amino FGs changed with time, pointing to a reorganization of the surface amines for the custom-made samples. Such a trend is not observed for the NC samples over a similar time period (SI, Fig. S11 and S13). Possible explanations could be a reduced stability of the amine multilayers of small SiO_2_ NPs with respect to amine loss and reorganization of the accessible surface amines, consistent with published results for aminated planar surfaces.^48^ Alternatively, this could be ascribed to the preparation workflow for the qNMR measurements as the precipitation of the smallest SiO_2_ NPs of this series is more challenging compared to larger SiO_2_ NPs. Such effects were not observed for the 100 nm custom-synthesized aminated NPs, which reveal no indication for amine loss over similar time periods. Such a reorganization of accessible amino FGs and loss of total amine amount could explain the differences in the qNMR results between NRC and BAM and may also account for the larger deviations of the qNMR data between BAM and NRC noticed in the first bilateral comparison of BAM and NRC for 20 nm aminated SiO_2_ NPs from NC.^27^

### XPS measurement of amine content in the near surface region

Next, BAM and NRC assessed the amount of surface amino FGs on the differently sized aminated SiO_2_ NPs with XPS for dried solid SiO_2_ NPs drop casted onto gold-coated substrates utilizing in house protocols (see SI, Section 6 on XPS for more details). With these complementary XPS measurements, which do not require a labeling step, we also aimed to assess the influence of sample preparation steps such as NP separation by centrifugation mandatory for qNMR.

**Fig. 6:**
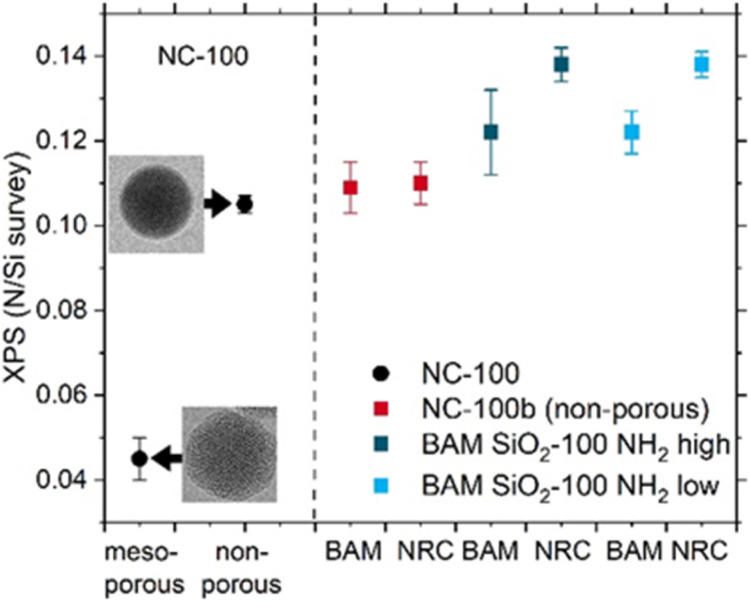
Overview of the XPS results from both labs obtained for the 100 nm particles. TEM images of the non-porous and mesoporous NPs are shown on left along with NRC Ni/Si ratio. The error bars reveal the standard deviations of measurements at three distinct points on the sample and are thus indicative of sample homogeneity.

The thereby obtained N/Si ratios are expected to reflect the amount of amine functionalization on the aminated SiO_2_ NPs in the near surface region with photoelectrons emitted from deeper regions in the material being attenuated by scattering processes. A comparison of the XPS data shown in Fig. 6 and Table S4 in the SI reveals the good agreement between the NRC and BAM data for most samples after taking into account the standard deviations for each lab, despite the use of different instruments and data evaluation procedures. At BAM, using a micro-focused XPS beam, a small decrease in N/Si ratio was observed with increasing radiation time, pointing to beam damage which may account for the lower value obtained for several samples (*e.g.*, BAM SiO_2_-100 NH_2_ high and low in Fig. 6).

Comparing the XPS data with the qNMR data and potentiometric titration results highlight some significant differences, particularly for the mesoporous NC-100 sample. Compared to the results derived from the other analytical methods, the XPS measurements considerably underestimate the actual amount of amino FGs, particularly for the mesoporous sample. Apparently, amine groups located on the inner surfaces of the mesoporous particles and not within the information depth of XPS of about 5 nm cannot be detected. Also, the XPS data reveal only a slightly higher amino FG amount for the BAM samples than for the NC-100 samples. This is consistent with a functionalization exceeding a monolayer as scattering processes can reduce the detection sensitivity of amines further away from the NP surface. In agreement with the results of the qNMR and potentiometric titration studies shown in Fig. 3, also XPS shows only minimal differences in amino FG amount between the two BAM SiO_2_-100 NH_2_ samples.

### Correlating XPS and qNMR measurements of amine content

**Fig. 7:**
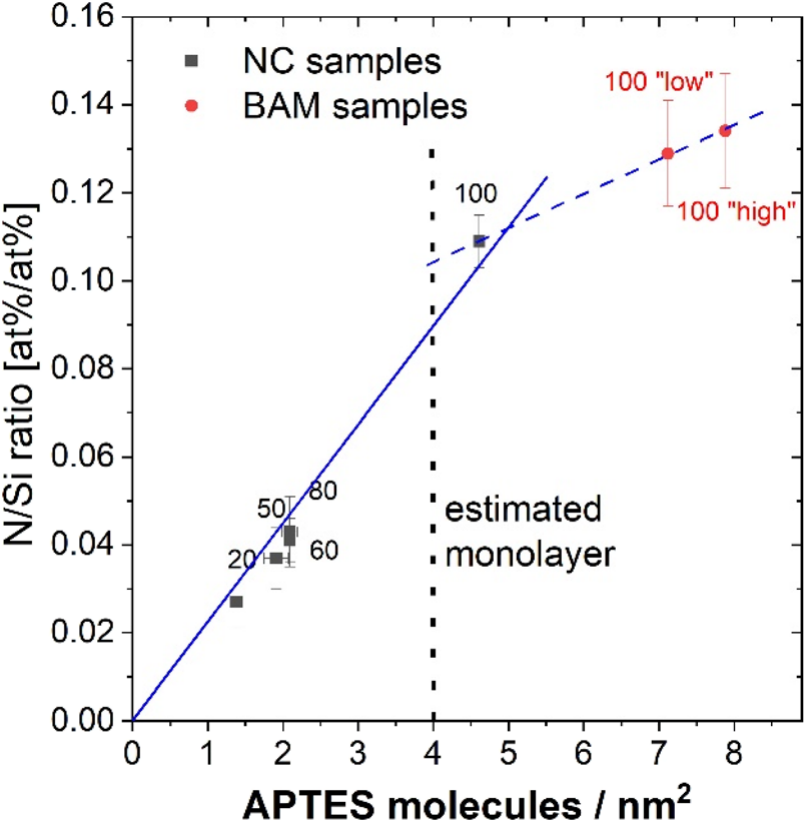
Correlation between the N/Si ratio detected with XPS and the number of APTES molecules per nm^2^ derived from the qNMR measurements showing the linear fits for both mono- and multi-layer regions of amino silane molecules. The size of the different samples is provided as numbers. The dashed line presents the theoretical value of 4 APTES molecules per nm^2^.

To correlate the XPS and qNMR results, we converted the amount of amine groups measured by qNMR to an areal density (number of APTES molecules per nm^2^). The specific surface area was estimated from TEM measurements which consume much less sample than BET. Therefore, a previously performed correlation between BET and TEM measurements for differently sized SiO_2_ NPs made by BAM was utilized, assuming similar densities of these SiO_2_ NPs and the NC samples. For these calculations and the data correlation displayed in Fig. 7, the means from the BAM and NRC results were used. The range was calculated from both series of measurements performed by NRC and by BAM with both methods. Fig. 7 suggests a linear correlation between the qNMR and XPS results, particularly for amine content up to a monolayer. This is consistent with a continuous growth of the APTES monolayer for the differently sized NC samples. For the BAM samples with a greater amount of amine groups, the slope of the line is significantly lower. This confirms the previous assumption that, in these samples, the amount of surface amino FGs exceeds a monolayer. For surface coverages of more than a monolayer, the N 1s XPS signal from the amino groups increases more slowly with the total number of FGs due to attenuating scattering processes. These results can be, however, also influenced by variations in particle size and shape as well as deviations from ideal APTES layer growth on the SiO_2_ particles.

### Screening SiO_2_ NP aging

Finally, BAM remeasured selected NC samples from the first bilateral comparison in 2021, which had been stored as air-saturated ethanolic dispersions in the refrigerator to examine and compare the applicability and information content of the different methods used in this study for stability studies of aminated SiO_2_ NPs (SI Section 7). We thereby focused on NC-20, NC-50, and NC-120 and a particle characterization by DLS and zeta potential measurements (SI, Fig. S22) followed by the determination of the amount of amino surface FGs by the Fluram assay, potentiometric back titration, and qNMR. These measurements revealed a strongly reduced amount of surface amino FGs detected by the different methods for the smaller sized particles, that seems to be particularly responsive to aging-induced changes of surface FGs. The similar trends of the latter two methods also indicate that protons can penetrate the silica network.

## Conclusions

Aiming to develop validated and eventually standardized methods for NM surface characterization and quantification of surface FGs with known uncertainties, we performed a multi-method characterization study on measuring surface amino FGs on structure-analytically well characterized silica nanoparticles (SiO_2_ NPs) with sizes of 20–100 nm. As a prerequisite for the broad applicability of our results, we studied large sets of representative custom-made and commercial aminated SiO_2_ NPs from different manufacturers, prepared with different commonly utilized sol–gel routes, different amounts of surface amino FGs, and different porosity. Our multi-method approach included an optical assay and a potentiometric back titration method as fast, cost-efficient, and automatable screening methods, that require the interaction of the FGs with a reporter, for signal generation and present ideal tools for quality and stability control, and more advanced solution qNMR. This traceable bulk method chemo-selectively applied by both NRC and BAM measures amines released from NMs that have been separated from SiO_2_ NPs dispersions by centrifugation, dried, weighed, and dissolved. qNMR measurements were complemented by both labs by XPS. These XPS measurements examined dried NM samples deposited on a solid support by drop casting of NM dispersions and exclusively provide information on near-surface FGs.

Specific aims of this study were to assess and fine-tune qNMR workflows to derive a protocol that gives good repeatability in each lab and good reproducibility between both labs and is applicable to different types of surface functionalized silica NMs. Such a protocol provides the basis for future interlaboratory studies with a number of partners as commonly required to provide per-normative data for method standardization. By comparing the results of the four analytical methods used in this study, providing different, yet closely connected measurands, we aimed to assess and identify method-inherent limitations. This included the influence of the size and spatial requirements of the signal-generating reporter, which can result in a reporter-specific underestimation of the FG amount, the level of chemo-selectivity, and the exclusive provision of information on near-surface FGs.

Our results indicate that bulk qNMR measurements with their inherent chemical selectivity, originating from the usage of selected NMR signals for FG quantification, can quantify all FGs present, *i.e.*, FGs at the particle surface, located within pores, and buried inside aged particles with chemically modified surface chemistries. Based on our comparative measurements, solution qNMR workflows could be further optimized and standardized, and method-inherent advantages and drawbacks could be identified. Critical for the accuracy and reliability of qNMR measurements are optimized sample preparation workflows and purification steps, removing unbound ligands or magnetic or paramagnetic species. Challenges include, *e.g.*, multilayers of amino silane molecules introduced by surface functionalization, known to be structurally fragile, which can be removed during solvent exposure or multiple washing steps or possibly partly removed by centrifugation at high speed. The latter is required for very small particles like SiO_2_ NPs with sizes <50 nm.^49^

Contrary to qNMR measurements of dissolved NMs, the applicability of optical assays that exclusively measure surface FGs by labeling with reporters with a certain size for signal generation is limited for determining and quantifying FGs located within the pores of (meso-) porous NMs. This was exemplarily demonstrated for the 100 nm mesoporous aminated SiO_2_ NPs for the Fluram assay and XPS. The good match between the results of the qNMR measurements and the less sensitive and selective potentiometric back titration underlines the applicability of this simple, cost-efficient, fast, and automatable electrochemical method for the screening and determination of surface amino FGs. Considering the different method-inherent sensitivities, the data determined for dispersed NPs match well, also for mesoporous SiO_2_ NPs and aged NC samples. This also highlights the advantage of ultrasmall reporters such as protons for signal generation compared to larger dye reporters, that apparently cannot penetrate silica networks. However, contrary to qNMR, the potentiometric back titration lacks chemo-selectivity regarding the source or origin of the respective (de)protonatable groups, here amino FGs. Therefore, the presence of impurities bearing (de)protonatable functionalities such as surfactants can result in an overestimation of the amount of amino FGs. In this respect, in-depth information on NM synthesis and surface functionalization can be helpful to limit or tackle such sources of uncertainty. The qNMR method is compatible with a wider range of FG structures and can also provide evidence for impurities. The relatively good correlation obtained for the qNMR and XPS data for nonporous surface-aminated SiO_2_ NPs, using qNMR data converted to an areal density, underlines the applicability of both methods for quantifying surface amino FGs on NMs. XPS modelling approaches will be used to facilitate a direct comparison of qNMR and XPS data that may yield traceable XPS measurements. This is, however, only applicable for FGs at the surface or near surface region as photoelectrons emitted from deeper regions in the material are increasingly attenuated by scattering processes.

Our muti-method characterization study elegantly demonstrates that correlating analytical methods providing different, but connected measurands, can be utilized to derive structure–property relationships for NMs, which are also advantageous for the design of sustainable and safer NM. As revealed in this study on aminated SiO_2_ NPs, XPS with its limited information depth and Fluram assays, requiring a chemical reaction with a relatively large reporter underestimate amine content when some of the amine groups are not “accessible”, *e.g.* in the case of mesoporous particles or for multilayer functionalization. Higher values are likely for chemo-selective qNMR and the potentiometric titration which responds to all FGs being protonatable. This order can be utilized to obtain information on, *e.g.*, surface morphology and FG accessibility as best demonstrated here for the characterization of the mesoporous aminated SiO_2_ NPs. The information derived from the Fluram assay on the amount of surface FGs still accessible for labeling reactions can be especially valuable for the stability monitoring and aging studies of surface engineered NMs intended for applications requiring successive labeling steps. This information cannot be easily extracted from the other analytical methods. For example, qNMR gives information on the stability of surface-aminated NMs in terms of total amine content, which is not available by the Fluram assay, that can, however, provide valuable insights into the rearrangement of surface amines and changes in the accessibility of amino FGs. For (de)protonatable FGs such as amino and carboxylate groups, electrochemical methods like potentiometric back titration can ideally complement optical assays to simplify and speed up FG screening, quality control monitoring and stability studies.

Finally, we compared the presented methods to other approaches that have been used in the literature for determining FG content. Elemental analysis, which has been employed in some cases does not contain information on the chemical and structural identity of FGs or impurities.^25^ Commonly employed thermogravimetric analysis can provide quantitative or semi-quantitative data on adsorbed coatings and FGs, but give little structural information unless combined with either mass spectroscopy or FT-IR and is not easily traceable.^12,25,49^ There is one study of silica NPs, a reasonably good agreement was obtained between the amine content measured by qNMR and TGA data.^25^ However, in contrast, earlier NRC results demonstrated that qNMR was generally a more useful quantitative tool than TGA especially for samples with relatively low FG content or high molecular weight FGs.^47,50^ This is primarily due to the loss of water over a wide range of temperatures, some of which overlap with FG loss for silica NPs and which require correction for water loss.

Overall, the validated measurement protocols and workflows present an important step to ease quality control of NM production and stability and to tailor NM functionality and safety. These measurement protocols also present an important basis for reliable and comparable toxicity and exposure studies with NMs. With such multi-method approaches and sets of surface-functionalized NMs, that are representative for commercial and custom-made samples, sources of uncertainty and method-inherent limitations can be effectively identified, and workflows optimized. To address the urgent need for standardized workflows and protocols for FG quantification, considering major sources of uncertainty such as sample preparation and data evaluation, in the near future, we plan to organize interlaboratory comparisons (ILCs) on qNMR and XPS with partners from metrology institutes and expert academic and industrial labs utilizing the optimized measurement protocols from this study.

## Author contributions

IT: conceptualization, investigation, experimental design, validation, visualization, writing – original draft, writing – review & editing; IR: investigation, experimental design, validation; MPT: investigation, validation; PCS: investigation, validation; OK: investigation, validation; GL: conceptualization, investigation, experimental design, validation, writing – review & editing; JR: conceptualization, investigation, experimental design, validation, visualization, writing – review & editing; LJJ: conceptualization, experimental design, validation, visualization, writing – original draft, writing – review & editing, resources, project administration; AB: conceptualization, experimental design, validation, resources, writing – review & editing; URG conceptualization, experimental design, validation, writing – original draft, writing – review & editing resources, project administration. All authors have approved the final manuscript.

## Conflicts of interest

There are no conflicts to declare.

## Supplementary Material

NA-OLF-D5NA00794A-s001

## Data Availability

The data supporting this article have been included as part of the SI. Supplementary information: SiO_2_ NP synthesis and characterization (DLS, NTA, Zeta Potential, TEM), and FG quantification are given in the SI. See DOI: https://doi.org/10.1039/d5na00794a.
