# Supplementary material for: Quantifying surface groups on aminated silica nanoparticles of different size, surface chemistry, and porosity with solution NMR, XPS, optical assays, and potentiometric titration
Source: Nanoscale Adv. 2025 Sep 10;7(21):6888–900. doi: 10.1039/d5na00794a (PMC12445366; doi:10.1039/d5na00794a)
Supplement: NA-007-D5NA00794A-s001 [file NA-007-D5NA00794A-s001.pdf]

Supplementary Information (SI) for Nanoscale Advances.
This journal is © The Royal Society of Chemistry 2025

Supporting Information

Quantifying Surface Groups on Aminated Silica Nanoparticles of Different Size, Surface Chemistry, and Porosity with Solution NMR, XPS, Optical Assays, and Potentiometric Titration

Isabella Tavernaro[a,*], Isabelle Rajotte[b], Marie-Pier Thibeault[b], Philipp C. Sander[a], Oltion Kodra[c], Gregory Lopinski[b], Jörg Radnik[e], Linda J. Johnston[b], Andreas Brinkmann[b,*], Ute Resch-Genger[a,*]

[a]Division Biophotonics, Federal Institute for Materials Research and Testing (BAM), Richard-Willstätter-Str. 11, 12489 Berlin, Germany; isabella.tavernaro@bam.de; ute.resch@bam.de

[b]Metrology Research Centre, National Research Council Canada, Ottawa, Ontario K1A 0R6, Canada; andreas.brinkmann@nrc-cnrc.gc.ca

[c]Clean Energy Innovation Research Centre, National Research Council Canada, Ottawa, Ontario K1A 0R6, Canada

[d]Division Surface and Thin Film Analysis, Federal Institute for Materials Research and Testing (BAM), Unter den Eichen 44-46, 12203 Berlin, Germany.

Index

1. Particle Synthesis and Surface Modification (BAM SiO_2 NPs NH_2) ... 2
1.1 L-Arginine Approach ... 2
1.2 Stöber Approach ... 2
1.3 Surface Modification with Amino FGs ... 2
2. Particle Characterization ... 3
2.1 Zeta Potential ... 3
2.2 Dynamic Light Scattering (DLS) ... 4
2.3 Nanoparticle Tracking Analysis (NTA) ... 6
2.4 Transmission Electron Microscopy (TEM) ... 7
2.5 Theoretical number of amino groups per monolayer ... 9
3. Screening of the accessible number of amino groups via a (semi-)automated optical assay. 11
4. Screening of the total number of amino groups via potentiometric back titration ... 13
5. Quantitative Nuclear Magnetic Resonance (qNMR) Spectroscopy ... 14
6. X-ray photoelectron spectroscopy (XPS) ... 21
7. Reassessing selected aminated SiO_2 NPs from the first ILC ... 23
***8.* References ... 24**

1. Particle Synthesis and Surface Modification (BAM SiO_2 NPs NH_2)

1.1 L-Arginine Approach

The synthesis of silica nanoparticles (SiO_2 NPs) using L-arginine as a catalyst involved a multistep synthesis approach based on a modified sol-gel process.[1, 2] Thereby, tetraethyl orthosilicate (TEOS, Sigma Aldrich, Germany), the silica source, was dissolved heterogeneously in a biphasic system consisting of cyclohexane (Labsolute, Th Geyer, Germany) and an aqueous solution of L-arginine (Carl Roth GmbH, Germany). The hydrolysis and condensation of TEOS resulted in the formation of amorphous, non-porous SiO_2 NPs with diameters ranging from 25 nm to 50 nm, depending on the reaction temperature and synthesis steps. 25 nm sized SiO2 NPs were obtained by a one-pot synthesis of TEOS (5.5 mL, 24.63 mmol) with 91 mg (0.52 mmol) of L-arginine at 65°C in a cyclohexane/water mixture (4.5 mL / 69 mL), while the larger particles were obtained by an additional regrowth step of the 25 nm sized particles at 70 °C for 24 h. The obtained particle dispersions were purified via dialysis against water for 24 h (4 L, water exchange after 1 h, 2 h and 4 h) using a regenerated cellulose membrane with a molecular weight cut-off (MWCO) of 10 kDa (Nadir®, Carl Roth GmbH, Germany) to remove unreacted materials and by-products. The particle dispersions were precipitated with acetone (Labsolute, Th Geyer, Germany), centrifuged at high speeds, and the supernatant was carefully decanted. The particles were redispersed and stored in absolute ethanol (EtOH, Labsolute, Th Geyer, Germany).

1.2 Stöber Approach

As a second route for synthesizing amorphous, non-porous SiO_2 NPs the well-known Stöber approach was used.[1, 3] Therefore, a reaction mixture containing of absolute EtOH, MilliQ-water, and ammonia (25%, abcr GmbH, Germany) was prepared and TEOS was added directly after the addition of ammonia. The reaction mixture was stirred at room temperature (r.t. = 23 °C) or slightly elevated temperatures for 20 h to 48 h. For instance, 25 nm sized particles were synthesized by adding 0.55 mL (2.5 mmol) of TEOS into a mixture of MilliQ-water/EtOH/ammonia (0.41 mL / 23.57 mL / 0.50 mL) and stirring at r.t. for 48 h, while for the larger particles the precursor ratio was changed to 0.41 mL of MilliQ-water, 22.55 mL of ethanol, 1.04 mL of ammonia and 1.05 mL (4.7 mmol) of TEOS at r.t. or higher reaction temperatures and a reaction time of 20 h. Depending on the obtained particle sizes the particle dispersions were either purified via dialysis against water for 24 h (4 L, water exchange after 1 h, 2 h and 4 h) or via centrifugation using an EtOH:MilliQ-water mixture. The particles with sizes of 50 and 100 nm were stored in EtOH, while the 25 nm particles were stored in MilliQ-water.

1.3 Surface Modification with Amino FGs

The synthesized SiO_2 NP were aminated with 3-aminopropyltriethoxysilane (APTES) in ethanol using a previously described method.[4] Thereby, the SiO_2 NPs were first dispersed in absolute EtOH or a EtOH:MilliQ-water mixture (9:1) and degassed under argon for 30 min at 30 °C, then adequate amounts of APTES (abcr GmbH, Germany) were added under gentle stirring. We chose a high, medium, and low FG density of APTES that exceeded a single monolayer for the grafting of the 25 nm, 50 nm, and 100 nm sized particles. Therefore, we calculated the amounts of APTES required for a monolayer and used a factor of 25.0 for a high density, 12.5 for a medium density and a factor of 5.0 for a low density of BAM SiO_2-25/50

NH_2. The utilized factor for BAM SiO_2-100 NH_2 low was 12.5 and for BAM SiO_2-100 NH_2 high was 25.0. The reaction mixture was stirred under argon atmosphere and slightly elevated temperatures for 2 d to finalize the hydrolysis of the ethoxy groups and allow the formation of the stable siloxane groups on the particle surface. Purification was done by precipitation via centrifugation and several washing steps using EtOH. The purification of the small 25 nm aminated SiO_2 NPs obtained by the Stöber approach (BAM SiO_2-25 NH_2 (Stöber)) via centrifugation was not possible without aggregation of the particles. Hence, the particle suspension was purified via dialysis against water and stored in water.

2. Particle Characterization

Commercial aminated SiO_2 NPs were obtained from NanoComposix (USA) as ethanolic suspensions (≈10 mg/mL), referred to in the following as "NC-particle size" samples. NanoComposix (NC) disclosed that the SiO_2 NPs were synthesized by the Stöber approach and surface modified with 3-aminopropyl groups in a post-synthetic grafting step. One bottle of 20 nm (lot number: JTW0207), 50 nm (lot number: LBE0060), 60 nm (lot number: JRO0164), 80 nm (lot number: SAM0152), and 100 nm (lot number: MPP0063) aminated SiO_2 NPs each were shipped to NRC and to BAM. Two additional bottles of the aminated 100 nm SiO_2 NPs with the same lot number (MPP0063) were shipped to NRC and to BAM after several months. Additionally, a 100 nm aminated mesoporous SiO_2 NP sample of NC-100 was provided by NC to NRC which was subsequently also measured by BAM after sample exchange between the two labs. For remeasuring aminated SiO_2 NPs from NC from the first bilateral comparison of NRC and BAM published in 2021, thereby assessing aging effects and the response and sensitivity of the different analytical methods explored in this study, 20 nm (lot number: JRC0486), 50 nm (lot number: MEL0032), and 120 nm (lot number: JEA0209) were used. The physicochemical characterization data provided by the supplier, including the particle number concentration, the mass concentration of silica, the z-average from dynamic light scattering, the mean diameter and surface area from transmission electron microscopy (TEM), and the surface charge as obtained by zeta potential measurements were used for a comparison with our results.

2.1 Zeta Potential

The surface modification of the silica nanoparticles (SiO_2 NP) with APTES was assessed via zeta potential measurements using a Zetasizer Nano ZS (Malvern Panalytical, Germany). All measurements were performed in triplicate at 25 °C in folded cuvettes (DTS-1070, Malvern Panalytical), dispersing all particles (0.2 mg/mL SiO_2 NPs) in 1 mL of MilliQ-water or EtOH. The zeta potential was calculated from the NP electrophoretic mobility using the Einstein-Smoluchowski theory. The NRC samples were measured after sample exchange with BAM during the ongoing qNMR study. The lower zeta potentials of these samples could provide a hint for NP aging affecting NP surface chemistry (Figures S1 and S2).

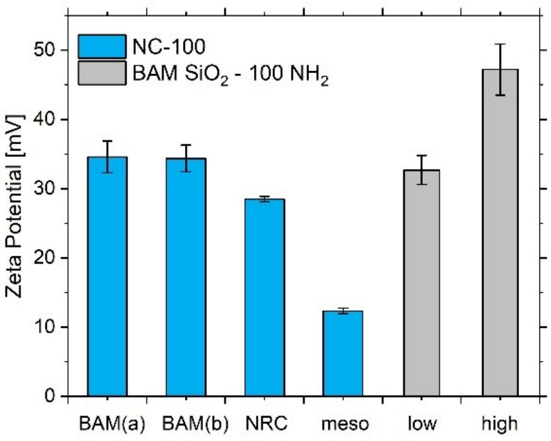

Figure S1. Overview of the zeta potential measurements for the different 100 nm sized particles performed at BAM. The results obtained for the NanoComposix (NC) samples NC-100 are shown in blue and the 100 nm sized custom-made particles synthesized by BAM are shown in grey. Low and high represent the different concentrations of APTES used for the surface modification steps. BAM(a), BAM(b), and NRC indicate the different bottles sent to the respective labs by NC. NC-100 (meso) is the mesoporous aminated SiO_2 NP sample. This morphology accounts for its relatively low zeta potential.

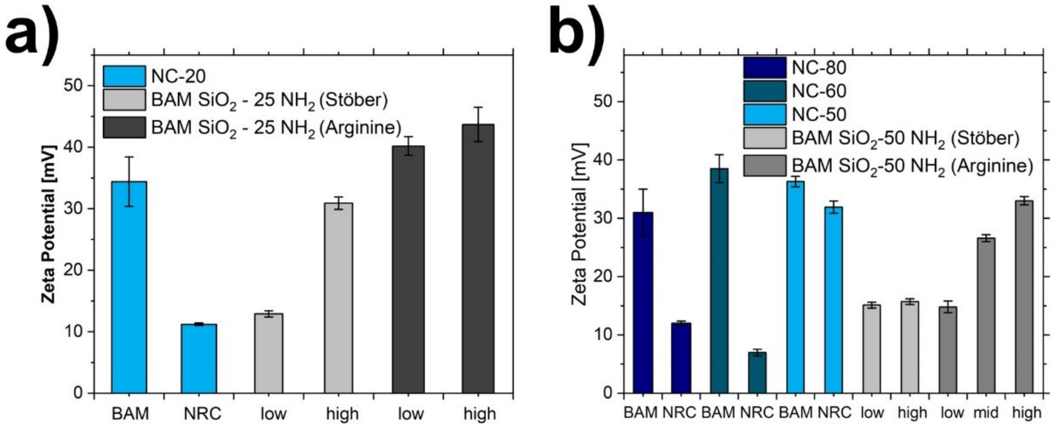

Figure S2. Overview of the zeta potential measurements of the small- and middle-sized particles. The results obtained with the NC samples NC-20, NC-50, NC-60, and NC-80 are shown in blue. The specifications BAM and NRC indicate the original bottles sent to the two institutes by NC. The original NRC samples were sent to BAM several months after receipt for additional measurements. The results of the measurements of the BAM-synthesized SiO_2 NPs BAM SiO_2-25/50 NH_2 (Stöber) and BAM SiO_2-25/50 NH_2 (L-arginine, referred to in the following as Arginine) are shown in grey. The specifications Stöber and Arginine refer to the different syntheses, while low, mid, and high represent the different concentrations of APTES used during the surface modification steps. All measurements were done by BAM.

2.2 Dynamic Light Scattering (DLS)

The average hydrodynamic particle diameter and the dispersity of the particle size were recorded with a Zetasizer Nano ZS (Malvern Panalytical, Germany) equipped with a 633 nm laser. For DLS measurements SiO_2 NPs were dispersed in MilliQ-water or EtOH with a particle concentration of 0.2 mg/mL. The samples were measured at a back scattering angle of 173° applying the cumulant method. The polydispersity index (PDI), intensity-based ($d_{h,i}$) and number-based ($d_{h,0}$) hydrodynamic diameter were calculated by the Zetasizer Software (Version 8.02) using a refractive index of 1.4649 for silica, a refractive index of 1.3300 and a viscosity of 0.8872 cP for water, or a refractive index of 1.361 and a viscosity of 1.0400 cP for

EtOH. Prior to the sample measurements the accuracy of the device was tested with a NIST-traceable particle size standard based on polystyrene nanoparticles (100 nm, PS-ST-B1261) from microparticles GmbH (Germany). All measurements were performed in triplicate. The NRC samples were measured after the sample exchange of the qNMR study and might show an influence of aging.

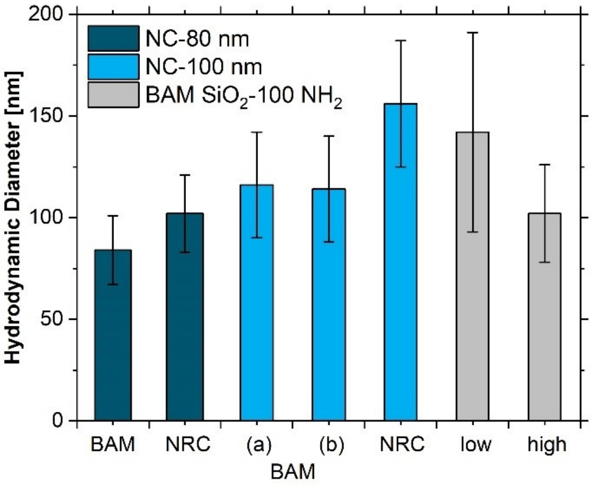

Figure S3. DLS results of the number-based hydrodynamic particle diameter ($d_{h,0}$) for the 80 nm and 100 nm sized particles. The NC samples NC-80 and NC-100 are shown in blue and the custom-made particles BAM SiO_2-100 NH_2 prepared with two different amino FG concentrations low and high are shown in grey. BAM (a), BAM (b), and NRC labels indicate the original bottles sent to the two labs by NC. The original NRC samples were sent to BAM several months after receipt for additional measurements. NC-100 (NRC) are mesoporous aminated SiO_2 NPs. The error bars indicate the size dispersity of the particles. All measurements were done by BAM.

For the presentation of the hydrodynamic diameters in Figure S3 and Figure S4 measured by BAM, we used intensity based DLS data converted to number-based distributions as a comparison of the results obtained for NTA and DLS is only possible for number-based hydrodynamic diameters ($d_{h,0}$). When calculating $d_{h,0}$ from the intensity-based hydrodynamic diameter ($d_{h,i}$) using assumptions from the Mie theory, this results in a higher uncertainty. However, the number-based hydrodynamic diameter could be also more accurate for the smaller-sized particles, because the number-distribution describes the relative proportion of multiple components in the sample based on their mass rather than based on their scattering (intensity of scattering is proportional to diameter^6 (Rayleigh approximation)).

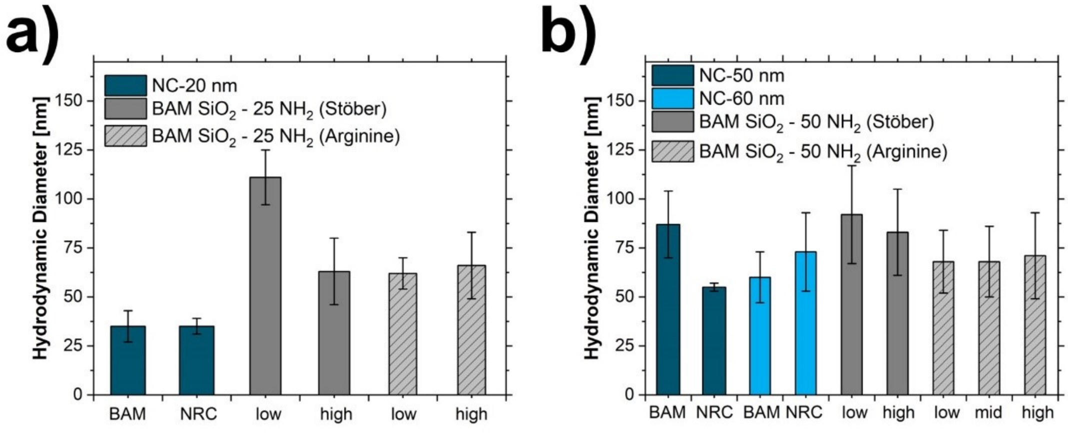

Figure S4. DLS results of the number-based hydrodynamic particle diameter ($d_{h,0}$) for the small- and middle-sized particles. The NC samples NC-20, NC-50, NC-60 are shown in blue, while custom-made particles synthesized by BAM (BAM SiO_2-25/50 NH_2 (Stöber) or BAM SiO_2-25/50 NH_2 (Arginine) are shown in grey. The different sol-gel routes used for NP synthesis by BAM, the Stöber and the L-arginine approach, are given in brackets. Low, mid, and high represent the different APTES concentrations used for the surface modification steps. The BAM and NRC labels below the blue bars indicate the original bottles sent to the the two labs by NC. The NRC samples were sent to BAM several months after receipt for additional measurements. The error bars indicate the size dispersity of the SiO_2 particles. All hydrodynamic diameters shown were measured by BAM.

2.3 Nanoparticle Tracking Analysis (NTA)

Due to the limitations of the technique, only aqueous dispersions of the larger 80 nm and 100 nm NPs were characterized by NTA at BAM to validate $d_{h,0}$ and the particle number concentration (PNC). The utilized NanoSight LM 10 system from Malvern Panalytical (Germany) was equipped with a 405 nm laser and the measurements were performed at a temperature of 25 °C, following the standards ISO19430 and ASTM E2834.[5] The NanoSight NTA software (Version: 3.32) was used to record 5 videos with 60 s and 25 fps for each of the highly diluted samples.

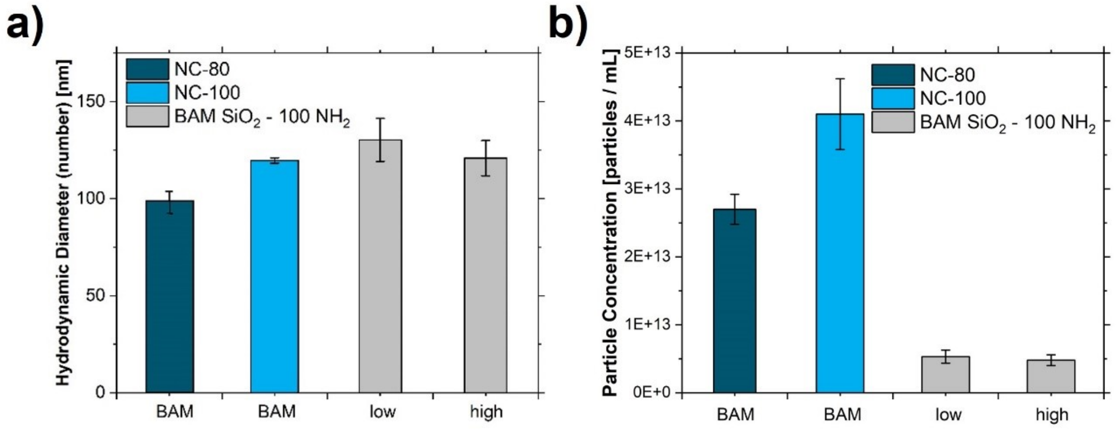

Figure S5. Results of the a.) particle size measurements and b.) particle number concentration for the larger sized particles using NTA. All measurements were done by BAM.

2.4 Transmission Electron Microscopy (TEM)

TEM micrographs were recorded with a Tecnai G2 20 S-Twin microscope (FEI Company, USA). Prior to the measurements, the particle dispersions were diluted 50-fold with EtOH or MilliQ-water and homogenized in an ultrasonication bath. Next 10 µL of the diluted NP dispersions were added dropwise onto TEM copper-grids (Plano GmbH, Germany) that were allowed to dry overnight. The NP size distribution was representatively determined for a randomly chosen sample of at least 150 particles using the X-ImageJ software (Version: 1.52 e). The images of the NC samples in Figure S9 were recorded to verify that the size data provided by NC were accurate.

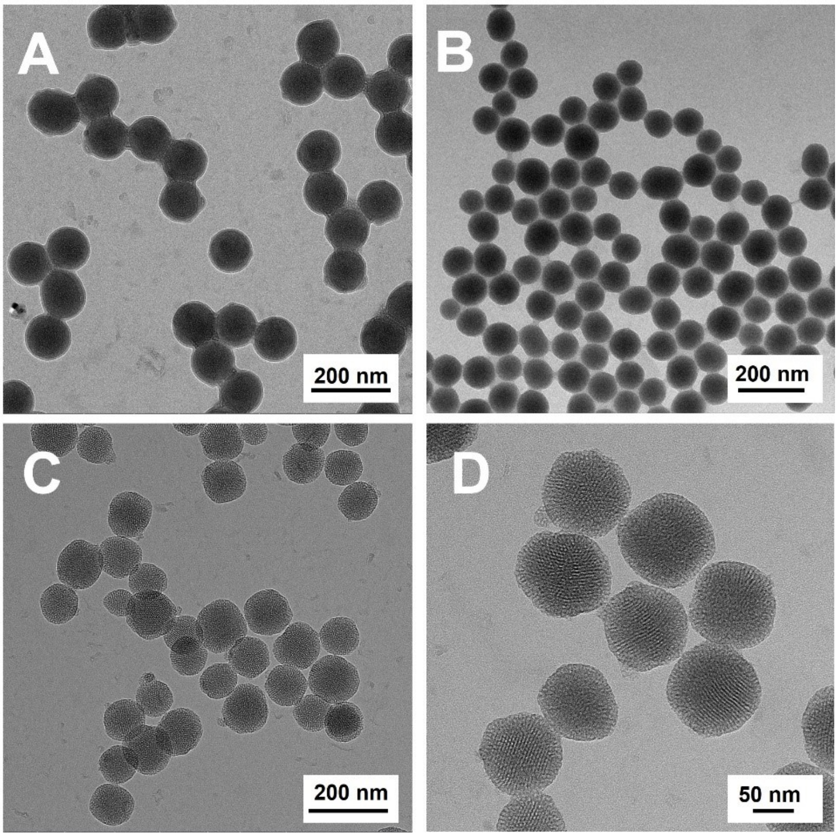

Figure S6. TEM Micrographs of the different 100 nm sized particles; A) NC-100b (non-porous), B) BAM SiO_2-100 NH_2 high, C) and D) NC-100 (mesoporous).

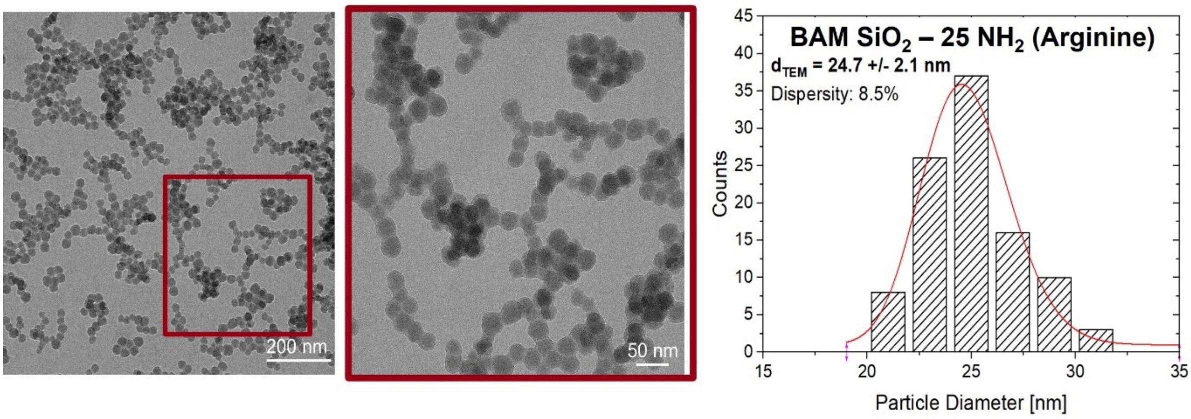

Figure S7. TEM micrographs and histogram of BAM SiO_2 – 25 NH_2 (Arginine).

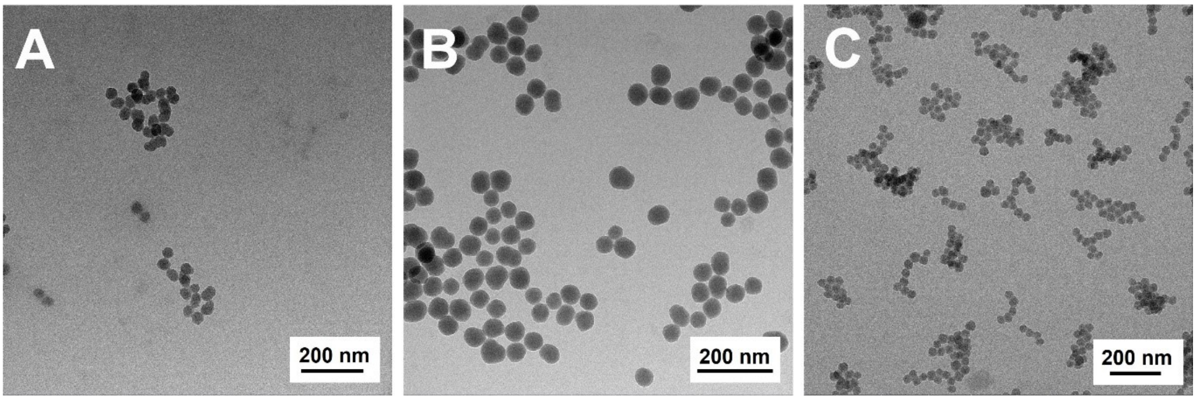

Figure S8. TEM Micrographs of the custom-made BAM SiO_2 NPs exemplarily shown for a high concentration of APTES used for surface grafting by BAM; A: BAM SiO_2-25 NH_2 (Stöber), B: BAM SiO_2-50 NH_2 (Stöber); BAM SiO_2-50 NH_2 (Arginine).

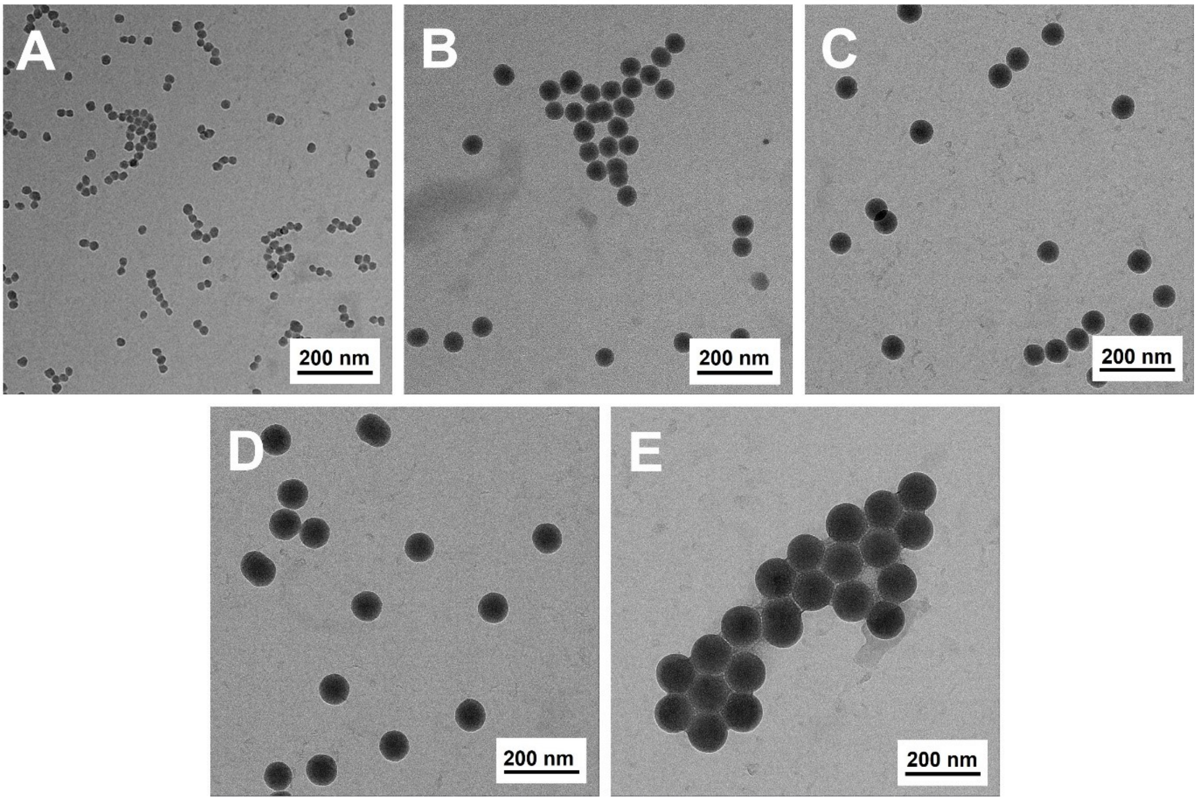

Figure S9. TEM micrographs of the commercial aminated SiO_2 NPs: A) NC-20 (BAM), B) NC-50(BAM); C) NC-60 (BAM), D) NC-80 (BAM), and E) NC-100a (BAM).

To determine the mass-related specific surface area of the differently synthesized BAM SiO_2 NPs the particle diameter obtained by TEM measurements and a silica density of 2.20 g/cm^3 for the custom-made particles obtained by the Stöber method or a silica density of 2.09 g/cm^3 for the particles synthesized by the L-arginine method were used in equation (1), that were taken from the literature.

Specific Surface Area: $SSA\ [m^2/g] = A / m = (4/3 \cdot \pi \cdot r^2) / (\delta \cdot 4/3 \cdot \pi \cdot r^3) = 6 / \delta \cdot d$ (1)

2.5 Theoretical number of amino groups per monolayer

The theoretical number of amino FGs per monolayer was estimated from the average particle radius obtained from TEM measurements, the SSA, and the silica density (ρ), using the equations (2) - (7). This approach is typically used in literature studies for silica NPs and by commercial NP supppliers.[4, 8, 9] We estimated 4 APTES molecules/nm², following the literature known average density of 2.1-4.2 molecules APTES per nm^2.[6] Note that this is consistent with hydroxyl contents that vary from 3 – 7 hydroxyls/nm^2 and with previous estimates of amines/nm.[8, 9, 10] Exemplarily shown are the calculations for NC-20 and the estimated monolayer coverage values are listed in Table S2:

Volume of a NP [m^3] = V = 4/3*π * r^3 = 4/3 * π (12.45 *10^{-9} m)^3 = 1.93 10 $^{-24}$ m^3 (2)

Mass of a NP [g] = $\rho(SiO_2)$ * V = 2200000 g/m^3 * 1.93 10^{-24} m^3 = 4.25*10^{-18} g (3)

Number of NP per mg = 0.001 g/ 4.25*10^{-18} g = 2.35*10^{14} (4)

Number of amino FGs per mg = 4*SSA = 4*1.09*10^{17} nm^2/mg = 4.4*10^{17} /mg
(5)

Number of amino FGs per NP = 4.4*10^{17} /mg /5.63*10^{13} /mg = 7782 (6)

Amount of APTES per mg = 4.4*10^{17} / 6.022*10^{23} = 730 nmol (7)

Table S1. Overview of the various particles and their intrinsic physico-chemical properties used in this study; *information taken from the NC homepage (lot number: CWW0151). For the NC samples and the BAM-SiO_2 NH_2 (Stöber), a density of 2.20 were used, while for BAM SiO_2 NH_2 (L-arginine) a density of 2.09 were used; n.d. = not determined.

Particle Name	d_{TEM} [nm]	$d_{h,0}$ (DLS) [nm]	$d_{h,0}$ (NTA) [nm]	PNC (NTA) [particles/mL]	PNC (weighted) [particles/mL]	Zeta potential [mV]	SSA [m^2/g]	NH_2 groups / monolayer
NC-100 (mesoporous)	108.3 ± 11.2	156 ± 31	n.d.	n.d.	6.84E+12	12 ± 1	629*	2.52E+18
NC-100 (non-porous)	106.8 ± 2.2 (a)<br>104.3 ± 2.5 (b)	114 ± 26 (a)<br>116 ± 26 (b)	120 ± 2 (a)<br>117 ± 2 (b)	6E12 ± 1E11 (a)<br>4E12 ± 5E11 (b)	7.14E+12 (a)<br>7.67E+12 (b)	35 ± 2 (a)<br>34 ± 2 (b)	25.5 (a)<br>26.2 (b)	1.02E+17 (a)<br>1.05E+17 (b)
BAM SiO_2- 100 NH_2 high	95.6 ± 6.3	142 ± 49	130 ± 11	5E12 ± 1E12	1.16E+13	47 ± 4	28.5	1.14E+17
BAM SiO_2- 100 NH_2 low	n.d.	102 ±24	122 ± 9	5E12 ± 1E12	1.16E+13	33 ± 2		
NC-80	83.9 ± 2.5	84 ± 17	98 ± 6	3E13 ± 2E12	1.47E+13	31 ± 4	32.5	1.30E+17
NC-60	64.4 ± 2.3	60 ± 13	n.d.	n.d.	3.25E+13	39 ± 2	42.4	1.69E+17
NC-50	55.8 ± 2.7	87 ± 17	n.d.	n.d.	5.00E+13	36 ± 1	48.9	1.96E+17
BAM SiO_2-50 NH_2 high (Stöber)	53.1 ± 4.2	92 ± 25	n.d.	n.d.	4.24E+13	16 ± 1	51.4	2.05E+17
BAM SiO_2-50 NH_2 low (Stöber)	n.d.	83 ± 22	n.d.	n.d.	4.24E+13	15± 1		
BAM SiO_2-50 NH_2 high (Arginine)	40.3 ± 2.7	68 ± 16	n.d.	n.d.	6.17E+13	33 ± 1	71.24	2.85E+17
BAM SiO_2-50 NH_2 mid (Arginine)	n.d.	68 ± 18	n.d.	n.d.	6.17E+13	27 ± 1		
BAM SiO_2-50 NH_2 low (Arginine)	n.d.	71 ± 22	n.d.	n.d.	6.17E+13	15 ± 1		
NC-20	24.9 ± 2.2	35 ± 8	n.d.	n.d.	5.63E+14	34 ± 4	109.5	4.38E+17
BAM SiO_2-25 NH_2 high (Stöber)	18.2 ± 1.3	63 ± 17	n.d.	n.d.	5.28E+14	31 ± 1	149.9	5.99E+17
BAM SiO_2-25 NH_2 low (Stöber)	n.d.	111 ± 14	n.d.	n.d.	5.28E+14	13 ± 1		
BAM SiO_2-25 NH_2 high (Arginine)	24.7 ± 2.1	66 ± 17	n.d.	n.d.	1.82E+14	44 ± 3	116.2	4.65E+17
BAM SiO_2-25 NH_2 low (Arginine)	n.d.	63 ± 8	n.d.	n.d.	1.82E+14	40 ± 2		

3. Screening of the accessible number of amino groups via a (semi-)automated optical assay

Prior to the qNMR studies, the amination of the SiO_2 NPs was assessed with an optical assay, which relies on the chemical reaction of the dye precursor 4′-phenylspiro[2-benzofuran-3,2′-furan]-1,3′-dione (fluorescamine, fluram®) with primary amino groups. This assay, which has been recently automated by us, is ideal for the screening of surface amino FGs.[4] For this optical assay, 1.51 µmol fluorescamine (abcr GmbH, Germany) in 15 µL of acetonitrile (Sigma Aldrich, Germany) was added to the previous prepared NP samples (0.2 - 0.5 mg), ethanolamine (EA) samples (Sigma Aldrich, Germany; used for the calibration curve), and the control samples, all in 1 mL of phosphate buffer (0.01 M, pH 8) using the Assist Plus automated pipetting system (INTEGRA Biosciences, Switzerland). After 45 min of incubation at room temperature (T = 23 °C), the samples were measured with an Infinite M200 pro microplate reader from Tecan (Switzerland), using an excitation wavelength (λ_{ex}) of 392 nm and an emission wavelength (λ_{em}) of 480 nm. The maximum amount of fluram reporter dye was estimated, using a size of 0.66 nm^2 for a fluram molecule and a monolayer of amino functional groups for the different sized particles. No steric hinderances and no electrostatic repulsion between the dye molecules were considered.

Table S2. Overview of the maximum amount of fluram reporter dye and amino FGs per monolayer.

Particle	Estimated amount of amino FGs for one monolayer [µmol/g]	Estimated maximum amount of fluram for one monolayer [µmol/g]
NC-100 (mesoporous)	4178	689
NC-100 (non-porous)	169	29
BAM SiO_2-100 NH_2	189	31
NC-80	216	36
NC-60	281	46
NC-50	325	54
BAM SiO_2-50 NH_2 (Stöber)	341	56
BAM SiO_2-50 NH_2 (Arginine)	473	78
NC-20	730	120
BAM SiO_2-25 NH_2 (Stöber)	995	164
BAM SiO_2-25 NH_2 (Arginine)	772	127

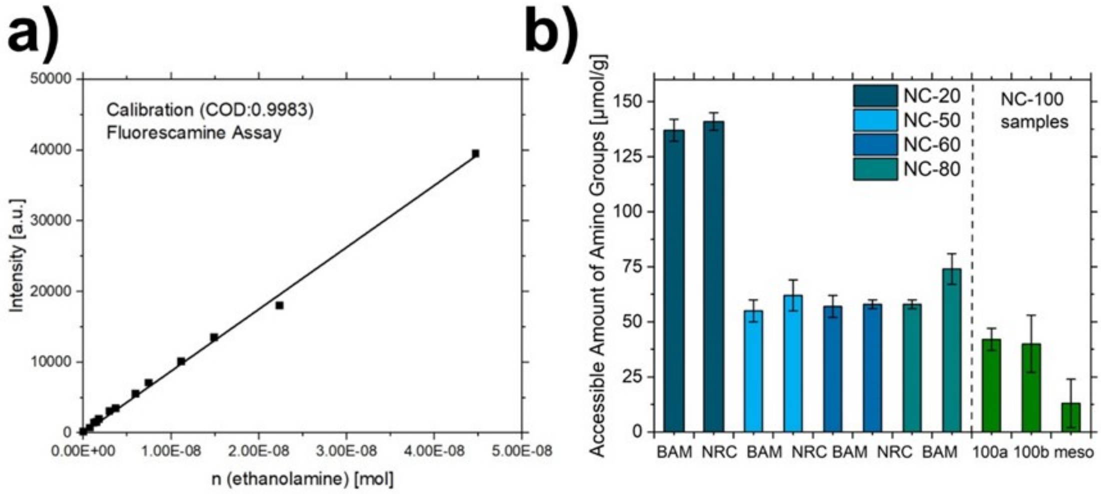

Figure S10. Overview of the fluorescamine (fluram) assay results for the bottle-to-bottle variability of the NC samples NC-20, NC-50, NC-60, NC-80, and NC-100 (100a and 100b = different bottles from BAM; meso = mesoporous sample) after the sample exchange and storage in ethanol over 14 months.

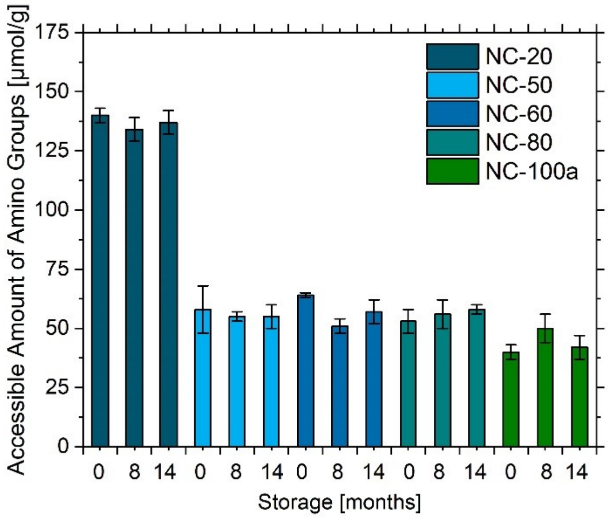

Figure S11. Overview of the results obtained with the fluram assay for the NC samples of the bottles sent to BAM after various storage times in ethanolic dispersion (10 mg/mL).

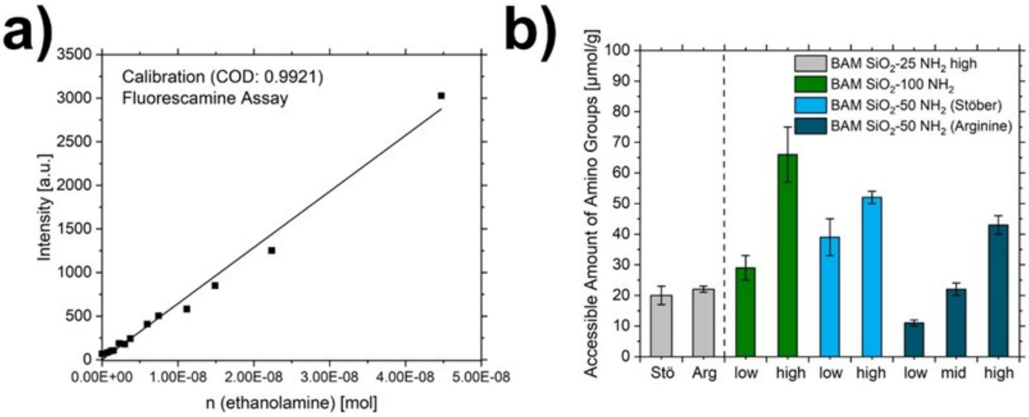

Figure S12. Overview of the fluram assay results obtained for different aminated SiO_2 NPs synthesized by BAM after storage in ethanolic dispersion (BAM SiO_2-50/100 NH_2) and in aqueous dispersion (BAM SiO_2-25 NH_2). Stö: Stöber method; Arg: L-arginine route; low, mid, and high are the APTES concentrations used for the postsynthetic grafting step.

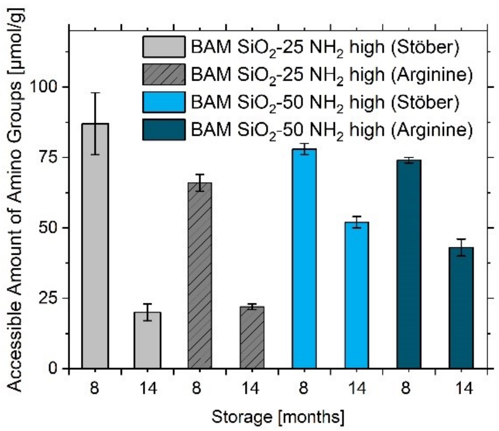

Figure S13. Overview of the results obtained with the fluram assay for the custom-made samples prepared at BAM after various storage times in aqueous dispersion (BAM SiO_2-25 NH_2 high) and ethanolic dispersion (BAM SiO_2-50 NH_2 high) with a particle concentration >10 mg/mL.

The variation observed in Figure S12 may suggest that the homogeneity of the functionalization with amino FGs on the surface of these SiO_2 NPs is quite variable. Overall, this may indicate that there are very different organizations of amino FGs and variations in the accessibility of these FGs for the samples with relatively high amine amounts. Measurements after several months of storage (Figure S11 and Figure S13) reveal that the storage dispersion and concentration of the particles have a high impact on the stability of the amino FGs.

4. Screening of the total number of amino groups via potentiometric back titration

Screening of the total number of (de)protonable FGs on the aminated SiO_2 NPs was performed by adapting a potentiometric back titration method previously described by us.[4] Therefore, the aminated SiO_2 NPs were dispersed in 1 mM hydrochloric acid (Carl Roth GmbH, Germany) and stirred only for 60-90 min at room temperature (T = 23 °C) to prevent potential hydrolysis

of the aminated SiO_2 NPs. In a next step, the particles were separated from the dispersion by centrifugation for 30 min at 16,000 rcf. The supernatant was collected and titrated with 1 mM sodium hydroxide solution (Carl Roth GmbH, Germany) until a neutral pH was reached. The pH values were determined using a SevenExcellence S475 pH meter from Mettler Toledo (Germany). Finally, the amount of the surface amino FGs was calculated from the difference of HCl concentrations. All supernatants showed a starting pH between 3.69 and 5.69, that ensured particle stability during incubation. The experiments were performed by two different operators (1 and 2) 8 months apart to test potential influences by particle aging and manual performance (Figure S14).

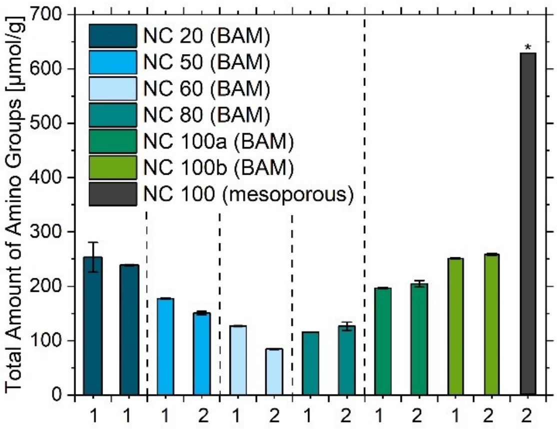

Figure S14. Overview of the potentiometric back titration results for the commercial NC samples performed by two operators (1 and 2) at BAM 8 months apart. The experiments were performed in triplicate except NC-100 mesoporous (* = single experiment).

5. Quantitative Nuclear Magnetic Resonance (qNMR) Spectroscopy

Workflows and protocols utilized for the qNMR measurements of NRC and BAM were stepwise fine-tuned, thereby starting, adapting and eventually standardizing the workflows and protocols developed for the first ILC to give a single protocol to be used by both labs.[7] Therefore, different samples of aminated SiO_2 NPs were employed. For iteration 1, with focus on particle amount, centrifugation, and drying steps, non-porous aminated 25 nm and 50 nm SiO_2 NPs with one amino FG density were used, prepared by BAM with the common Stöber and the underexplored L-arginine method (BAM SiO_2-25/50 NH_2), and the protocols developed for our previous ILC. For iteration 2, focusing on the evaluation of the NMR spectra, new batches of similar non-porous aminated 25 nm and 50 nm SiO_2 NPs were prepared by BAM by amination of the same silica cores as used for iteration 1 to circumvent possible aging induced changes in surface amino FG amount. To study a potential influence of the degree of amination, different amounts of APTES were used during the postsynthetic grafting step to obtain particles with low and high NH_2 densities. In addition, 100 nm non-porous SiO_2 NPs with two different degrees of amination (BAM SiO_2-100 NH_2 low/high) were synthesized by BAM. In the third iteration, the optimized protocol was examined using non-porous animated SiO_2 NPs from the supplier NC, who also provided the samples for the previous ILC, with sizes of 20, 50, 60, 80, and 100 nm (NC-20, NC-50, NC-60, NC-80, NC-100). A sample of 100 nm mesoporous aminated SiO_2 NPs was also examined. A bottle of each size originating from the same batch, was sent by NC to each partner. In the final iteration 4, the original SiO_2 NP samples from NC were exchanged between BAM and NRC and then remeasured with the optimized protocols

together with a new 100 nm aminated SiO_2 NP sample from NC for protocol validation. The various factors that were optimized at each stage are summarized below.

5.1 Centrifugation/weighing/drying steps and amount of silica

Initial experiments used the best-practice protocols from each lab to assess amine content for 25 and 50 nm NPs prepared at BAM using the Stöber and L-arginine synthesis method. This gave the results shown in Figure S15. These experiments showed a larger than expected variation between labs. A control experiment at NRC tested for amine content of the supernatant since the NRC protocol did not involve a centrifugation step to remove any amine released from the silica (Figure S16). The results indicated a minor loss of amine (7%) from the 25 nm particles synthesized by the arginine method and a large amine loss (44%) from the 25 nm Stöber NPs which had been stored in water. There was <1% amine detected in the supernatant for the BAM SiO_2-50 NH_2 (Stöber). This loss of surface amines might be explained by insufficient purification after synthesis or through hydrolysis of the FGs from the surface during storage or transport of the sample. This finding can be also relevant for other surface modified NMs with non-covalently bound surface ligands or surface ligands that can be easily removed from the particle surface. As a result, it was decided to adopt a procedure that involved centrifugation of the sample, removal of supernatant from the pellet and drying prior to hydrolysis using strong base. Particles stored in water were also excluded from consideration due to concerns with their stability.

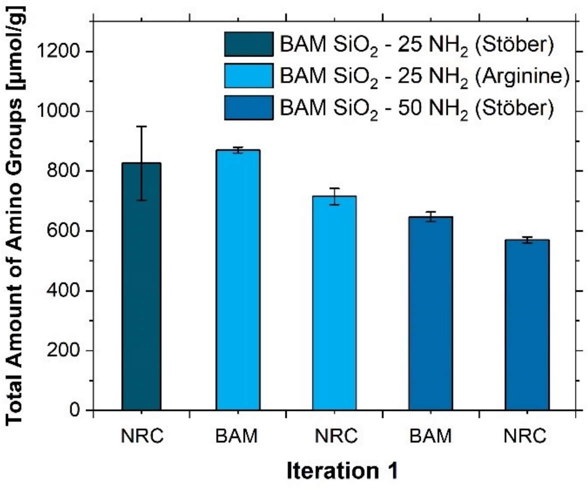

Figure S15. Overview of qNMR results obtained in iteration 1.

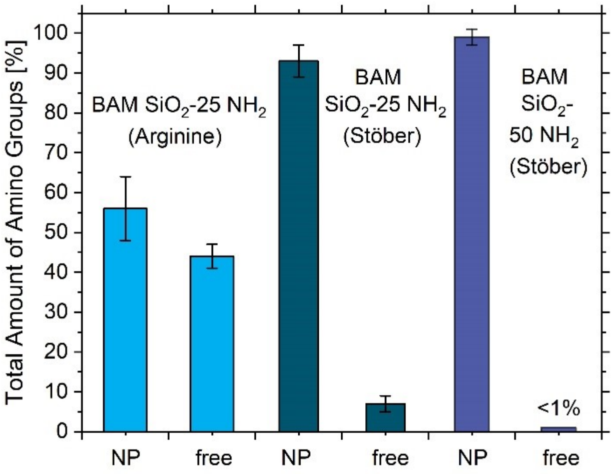

Figure S16. Potential influence of a centrifugation step followed by removal of the supernatant to separate amines that are noncovalently bound to the surface of the SiO_2 NPs or released by hydrolysis from the surface.

The mass loss due to drying or centrifuging of the microcentrifuge tubes prior to the experiment was also examined. The mass loss after drying was typically approximately 0.1 mg, and the drying step was therefore incorporated in the protocol. The weighing steps were performed with an Ultra Micro Balance XP-6U (Mettler Toledo, Germany) at NRC and a Cubis MCM 6.7 (Satorius, Germany) Ultra Micro Balance at BAM. The recommended silica mass was ~12 mg based on the amount used in the first comparison and a comparison of results using 10 and 20 mg samples (Figure S17). The latter showed minimal difference in qNMR results, consistent with earlier tests that varied the sample mass and base concentration.[8]

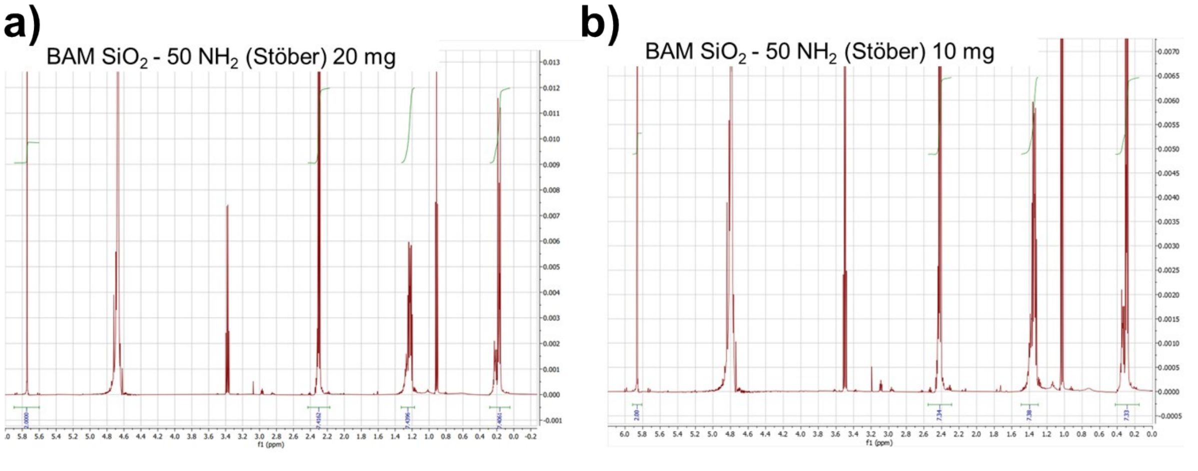

Figure S17. Potential influence of the used amount of sample per measurement, exemplarily shown for BAM SiO_2 –50 NH_2 (Stöber). A lower mass amount of 10 mg did not negatively influence the amount of amino FGs (a) 1111 µmol/mg and b) 1120 µmol/mg). As stated in the Experimental section of the MS, only the signals for the two CH_2 groups at 2.50 and 0.38 ppm were used for the reported data. The signal at 1.3 ppm was rejected for quantification due to its proximity to the methyl signal of residual ethanol.

5.2 qNMR data acquisition

The NMR data acquisition parameters were standardized to a number of 32 or 64 scans with a pulse angle of 90°, a pulse delay of 50 s, as well as an acquisition time of 3.60 s with a spectral width of 30 ppm at BAM, and acquisition times of 8.3 s and 5.5 s together with spectral

widths of 20 ppm and 30 ppm, respectively at NRC. pulse delay of 50 s. A JEOL ECZ-600 (600 MHz) NMR spectrometer (BAM) and a Bruker 400 MHz NMR (NRC) were used.

5.3 qNMR data processing

Freshly prepared custom-made particles (BAM SiO_2 NH_2) with sizes of 25 nm, 50 nm, and 100 nm, as well as particles with different FG densities were assessed by qNMR in both labs. The initial variation between labs was significant for some samples and the integration procedures were examined and standardized. BAM performed the data processing twice (Figure S18), one time analyzed using the protocol from BAM (BAM prior) and one time analyzed using the protocol from NRC (BAM after). A combined protocol for data processing was established, ensuring that satellites of the signals due to the amine aliphatic CH_2 groups at 2.50 and 0.38 ppm were included in the integration for FG quantification. However, the modified data evaluation did not significantly improve the comparability of the qNMR data and the size of the RSDs for smaller SiO_2 NPs.

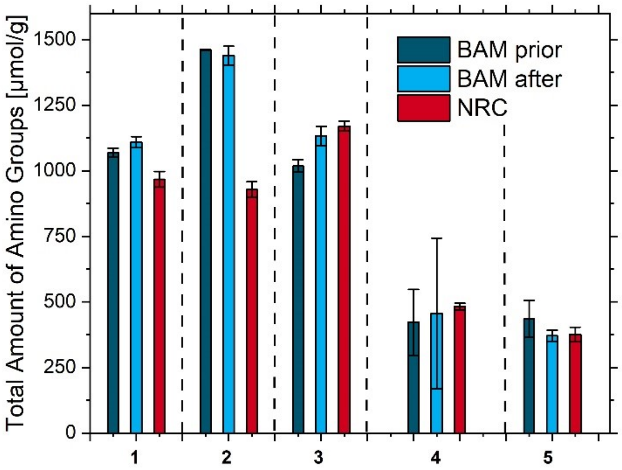

Figure S18. (1) BAM SiO_2 -25 NH_2 (Arginine); (2) BAM SiO_2 -25 NH_2 (Stöber); (3) BAM SiO_2 -50 NH_2 (Arginine); (4) BAM SiO_2 -50 NH_2 (Stöber); (5) BAM SiO_2 - 100 NH_2

5.4 Potential analyst effects

The comparability of the qNMR data and RSDs were examined for a new set of samples, non-porous animated SiO_2 NPs covering the same size range, i.e., NC-20, NC-50, NC-60, NC-80, and NC-100(a) as well as 100 nm mesoporous aminated SiO_2 NPs, all from NC, employing the previously optimized sample preparation, measurement, and data evaluation procedures. The optimized protocol considerably improved the comparability of the qNMR data and reduced RSDs. To study a potential operator influence, the data analysis of the qNMR experiments at NRC were performed by two operators, i.e., operators 1 and 2. The results indicated that the amine content was the same within the standard deviations of the analysis for each sample (Figure S19).

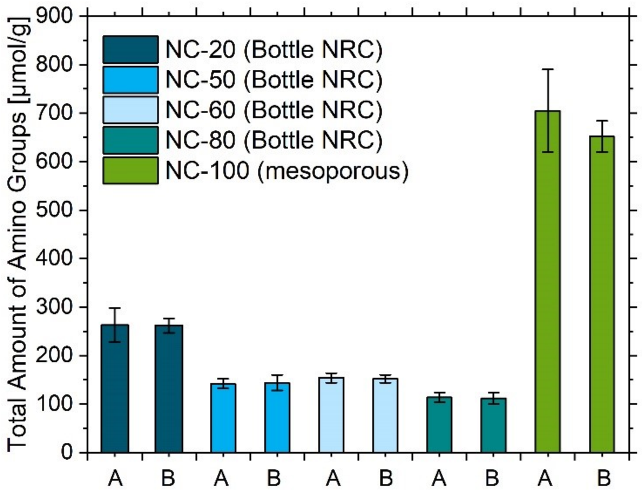

Figure S19. Overview of qNMR results obtained for the commercial NC samples, performed by two operators at NRC.

5.5 Stability of NC samples

To validate the optimized qNMR protocols, the SiO_2 NP samples were finally exchanged between NRC and BAM and remeasured together with a new sample of 100 nm aminated SiO_2 NPs (NC-100 (b)) from NC. The good match of the resulting qNMR data and the reduced RSDs confirmed the improvements in qNMR workflows and protocols (Table S3). The good comparability of the qNMR data of NRC and BAM obtained for the mesoporous aminated SiO_2 NPs, bearing a much higher amount of surface amino FGs exceeding that of non-porous aminated SiO_2 NPs of similar size covered with a monolayer of an amino silane by factors of about 3.5 underlines the advantage of bulk measurements of dissolved samples. In addition, the stability of a different 50 nm NC sample used for initial experiments was tested by remeasuring the sample after 14 months (Figure S20). the amine content was unchanged over this time period.

Table S3. Overview of the results obtained by qNMR at NRC and BAM by sample exchange between labs; the samples were measured shortly after receipt from NC or approximately 8-12 months later after sample exchange between the two labs; *= one replicate only.

Sample	BAM		NRC	
	Bottle BAM [µmol/g]	Bottle NRC [µmol/g]	Bottle BAM [µmol/g]	Bottle NRC [µmol/g]
NC-100 (mesoporous)	700 ± 13		705 ± 43	
NC-100a (non-porous)	187 ± 19	n.d.	191 ± 17[a] (194*)	n.d.
NC-100b (non-porous)	199*	n.d.	n.d.	202*
NC-80	103*	111 ± 7	109*	114 ± 5
NC-60	137*	149 ± 3	148*	154 ± 5
NC-50	168 ± 11	99*	138	142 ± 5
NC-20	238 ± 27	163 ± 15	215	263 ± 18

[a]6 months after receipt at BAM; at 8 months a single replicate gave 194 µmol/g

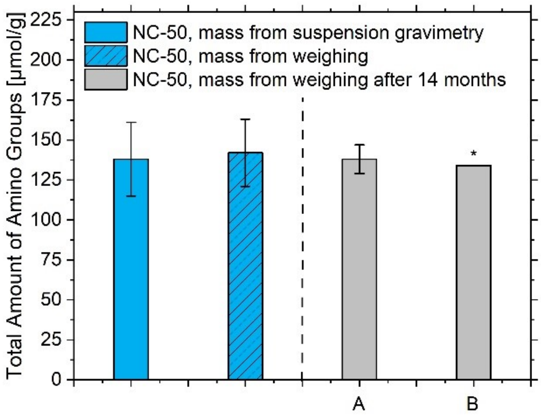

Figure S19. Overview of qNMR results for a NC-50 test sample that was measured initially with the sample mass obtained both by gravimetry of the stock suspension and the new protocol in which the sample was centrifuged, the supernatant removed, dried and weighed (blue bars). The sample was remeasured after 14 months using the new protocol by two analysts (A and B, grey bars); * = single experiment.

5.6 Final qNMR protocol

Using the results of the different iterations, a combined protocol was established for the bilateral comparison of qNMR performed by BAM and NRC. Freshly prepared samples were used. All experiments were performed in triplicate, unless otherwise noted.

BAM/NRC: Prior to the qNMR experiments, the mass concentration of the particle suspension was determined. Therefore, empty centrifuge tubes or vials were dried in the oven at 110 °C overnight. After cooling to room temperature, a specific volume of the particle suspension was added into each tube/vial and dried again in the oven at 110 °C overnight. The tubes/vials were weighed, and the mass concentration (mg/mL) was calculated. For the qNMR experiments, fresh centrifuge tubes were dried overnight in an oven at 100 °C and weighed afterwards. Next 1.0 mL of particle suspension was added in the pre-dried, pre-weighed centrifuge tubes, and the samples were centrifuged for at least 10 min at a minimum speed of 17,000 rcf. The supernatant was removed carefully without disturbing the pellet of silica particles. Depending on the concentration determined prior, these steps were repeated until approximately 12 mg of particles were in each tube. The centrifuge tubes were placed in the oven at 100 °C overnight to dry the pellet of particles. After cooling to room temperature, the tubes were weighed, and the mass of the particles was calculated. Next 800 µL of 1 M NaOD in D_2O was

added to each tube. The tubes were heated and agitated at 50 °C for a minimum of 3 h, before 50 µL of a maleic acid solution (10 mg/mL) as internal standard was added gravimetrically to each tube. The procedure for adding maleic acid was different for the two labs.

a) NRC: The filled, gastight syringe (250 µL) was weighed and 50 µL of the internal standard solution was dispensed into the tube. The syringe was weighed, and the added concentration was calculated from the weight loss.

b) BAM: 50 µL of the internal standard solution was added with a pipette to the tube, the change of weight was documented, and the added concentration was calculated.

The samples were transferred to NMR tubes and analyzed at BAM with an JEOL ECZ-600 600MHz NMR spectrometer and the MestReNova x64 software (Version: 14.2.1-27684) and at NRC using a Bruker 400 MHz NMR spectrometer with TopSpin 4.4.1 software. NMR parameters are 32 or 64 scans, sweep width of 20 or 30 ppm and a pulse delay of at least 50 s.

After Fourier transform (ef) was performed and the spectrum was manually phased for pure absorption mode, the chemical shifts were adjusted to the solvent signal, which was controlled against reported data. The baseline was corrected across a signal of interest by subtraction of a polynomial of the form $A + Bx + Cx^2 + Dx^3 + Ex^4$ using careful manual fitting. Next, the signals of the calibrant and analyte were integrated, taking care to choose the integral regions wide enough to contain the adjacent ^{13}C satellites. If possible, all signals of interest should be integrated. The NMR signals at 2.50 ppm and 0.38 ppm originating from the aliphatic CH_2 groups of the 3-aminopropyl groups were read out, while the signal at 6.30 ppm (2H) of the maleic acid was used as internal standard. Errors for BAM data are the standard deviations for multiple replicates and for NRC the standard deviations and additional sources of uncertainty have been included.

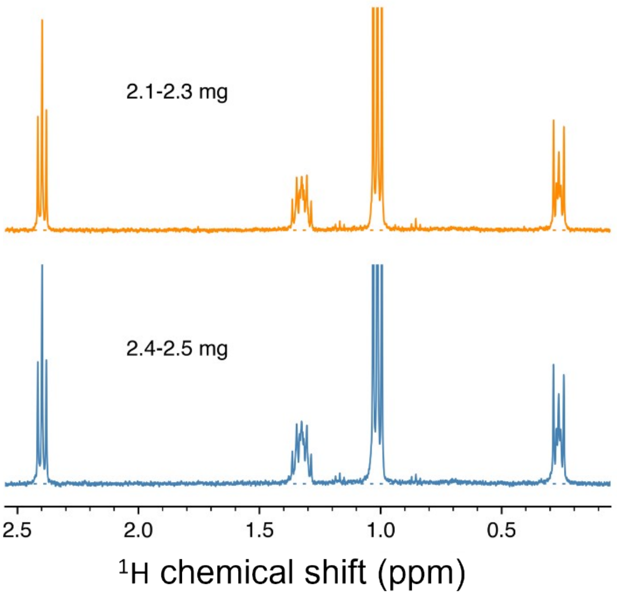

5.7 Representative qNMR spectra

Figure S21. qNMR spectra for BAM SiO_2-100 nm high and BAM SiO_2-100 nm low.

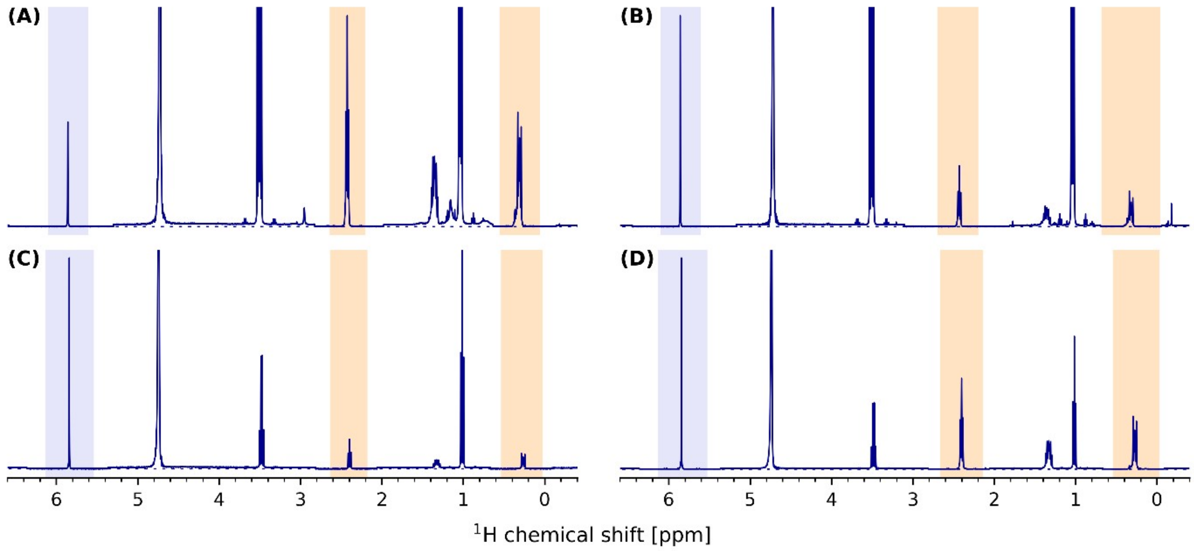

Figure S22. qNMR spectra recorded at NRC for 4 samples. Nanocomposix samples NC-50 (A) and NC-100 (B) and BAM SiO_2 100 NH_2 high (C) and BAM SiO_2 50 NH_2 high (Stober). The integrated analyte signals are highlighted in orange and the calibrant, internal standard in blue.

6. X-ray photoelectron spectroscopy (XPS)

XPS measurements of selected aminated SiO_2 NP samples were performed by NRC and BAM utilizing independent in-house sample preparation, measurement, and data evaluation protocols. XPS measures the atomic composition of the aminated SiO_2 NPs, deposited on a solid support, in the near surface region, with the effective probing depth determined by the escape depth of the photoelectrons (about 5 nm for the photoelectron energies used here) and can be hence used for quantifying the FG amount on NMs for elements such as sulfur, nitrogen, or halogens.[10] Typical sources of uncertainty include sample preparation steps, possible beam damage, spectrometer transmission function corrections, background subtraction and relative sensitivity factors used to extract atomic composition.[11] The XPS workflows used by NRC and BAM are described below, that varied only in the instrumentation. A comparison of the nitrogen-to-silicon ratios derived from survey and high-resolution measurements is shown in Table S5 and Figure S21 reveals a good match of the data for all samples within the SDSs except for the 80 nm samples.

The samples for XPS measurements were prepared at BAM and NRC by drop-casting on Au-coated substrate which is described elsewhere.[12] NRC used Au-coated glass as substrate, while BAM used Au-coated silicon wafers. At BAM the Au-coated Si wafers were cleaned with isopropanol, ethanol, and by ozone treatment. The wafers were not treated with the alkali glass cleaning solution to avoid any damages to the Au coating. At NRC, the Au-coated glass substrates were UV ozone cleaned for 30 minutes. After the ozone cleaning the SiO_2 NPs dispersion were deposited repetitively on the substrate with intermediate drying until the particle layer covered the surface well, as assessed by visual inspection.

At BAM XPS measurements were performed with a Quantes photoelectron spectrometer manufactured by Ulvac-PHI (Chanhassen, MN, USA). XPS spectra were recorded using monochromatized aluminum K_α radiation for excitation, at a pressure of approximately $5 \cdot 10^{-9}$ mbar. The electron emission angle was 45° and the source-to-analyzer angle was 45°. The binding energy scale of the instrument was calibrated following a PHI procedure which uses ISO 15472 binding energy data. The Si wafers were fixed on a double-adhesive carbon tape. Charge neutralization was performed with low-energy electrons and Ar^+ ions. C 1s at 284.8 eV

was used for the charge correction. For peak fitting a sum Gaussian-Lorentzian function was used. A Shirley background was used for the background correction.

At NRC an Axis UltraDLD spectrometer (Kratos Analytical, Manchester, UK) with monochromatized aluminum K_{α} radiation for excitation was used. The angle between the source and spectrometer is 60° and the samples were oriented normal to the spectrometer for a take-off angle of 90°. Three distinct areas (300 x 700 μm) were measured for each sample. The spectrometer pass energy was set at 80eV for the survey scans and 20 eV for high resolution scans. Data analysis was carried out using CasaXPS software (Casa software, Teignmouth, UK). Atomic compositions were determined by using the area under each peak after subtracting Shirley or linear backgrounds, and applying Kratos relative sensitivity factors.

Representative XPS spectra are shown in Figure S23 and results of survey scans are shown in Figure S24. All data recorded in both labs is summarized in Table S4.

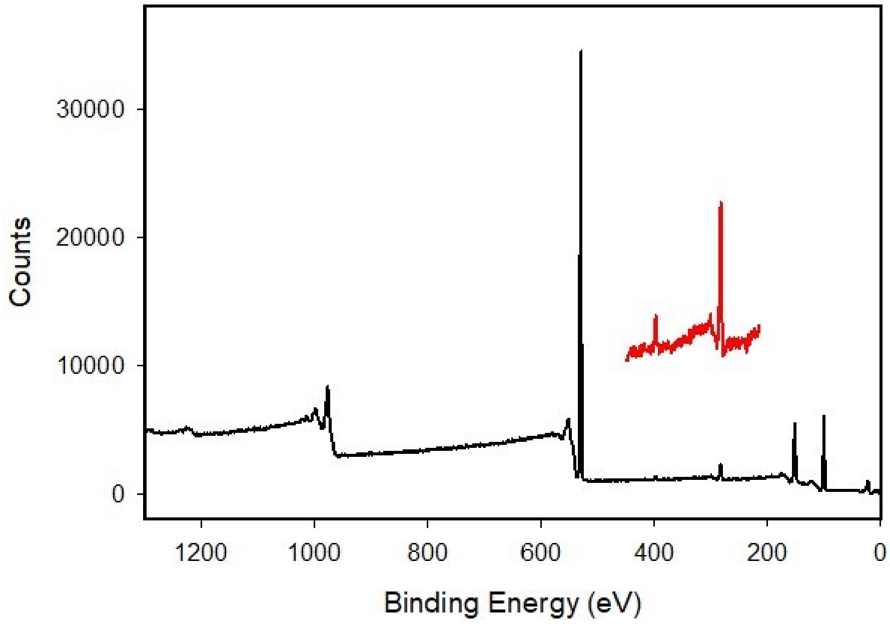

Figure S23. Representative XPS survey scan for NC 60 nm aminated silica nanoparticles obtained at NRC. The region with the N1s and C1s peaks has been multiplied by 10 to allow the signals to be more clearly seen. The peak areas are used to calculate the atomic % for each element, based on the tabulated sensitivity factors for the Kratos instrument used to obtain the spectrum.

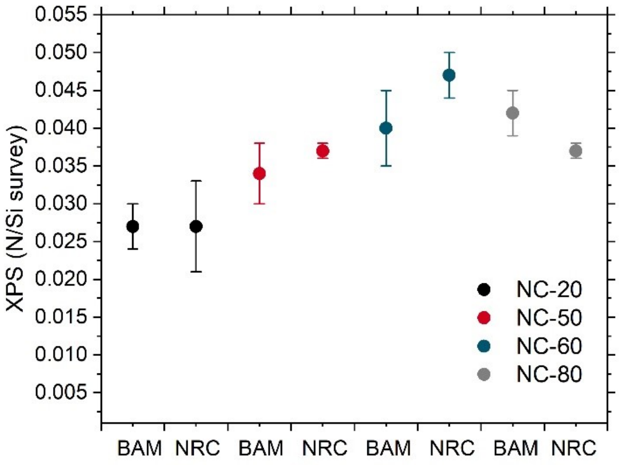

Figure S24. Comparison of the XPS measurements (survey N/Si) for the NC samples performed at BAM and NRC.

Table S4. Overview of the results obtained by the bilateral comparison of XPS measurements.

Sample	XPS measurements BAM		XPS measurements NRC	
	N/Si survey	N/Si high res	N/Si survey	N/Si high res
BAM SiO_2-100 NH_2 low	0.122 ± 0.005	0.095 ± 0.007	0.138 ± 0.003	0.116 ± 0.005
BAM SiO_2-100 NH_2 high	0.122 ± 0.010	0.091 ± 0.002	0.138 ± 0.004	0.109 ± 0.022
NC-100a (BAM bottle)	0.105 ± 0.002	0.138 ± 0.003	0.126 ± 0.006	0.079 ± 0.004
NC-100b (BAM bottle)	0.109 ± 0.006	0.109 ± 0.006	n.d.	n.d.
NC-100b (NRC bottle)	n.d.	n.d.	0.110 ± 0.005	0.081 ± 0.002
NC-100 mesoporous	n.d.	n.d.	0.045 ± 0.005	0.035 ± 0.003
NC-80 (BAM bottle)	0.042 ± 0.003	0.039 ± 0.004	0.037 ± 0.001	0.030 ± 0.001
NC-80 (NRC bottle)	n.d.	n.d.	0.046 ± 0.010	0.033 ± 0.001
NC-60 (BAM bottle)	0.040 ± 0.005	0.034 (1 area)	0.047 ± 0.003	0.028 ± 0.001
NC-60 (NRC bottle)	0.040 ± 0.006	0.028 ± 0.001	0.046 ± 0.002	0.033 ± 0.001
NC-50 (BAM bottle)	0.034 ± 0.004	0.027 ± 0.003	0.037 ± 0.001	0.024 ± 0.001
NC-50 (NRC bottle)	0.040 ± 0.006	0.025 ± 0.002	0.045 ± 0.003	0.031 ± 0.001
NC-20 (BAM bottle)	0.027 ± 0.003	0.022 ± 0.002	0.027 ± 0.006	0.022 ± 0.001
NC-20 (NRC bottle)	0.034 ± 0.004	0.024 ± 0.001	0.030 ± 0.003	0.23 ± 0.001

7. Reassessing selected aminated SiO_2 NPs from the first ILC

BAM remeasured 20 nm, 50 nm, and 120 nm non-porous aminated SiO_2 NPs from NC jointly explored by NRC and BAM in our first ILC[7] with the aim to gain information on aging-induced changes in amino FG amount, using the fluram assay, qNMR, and our validated potentiometric titration method. These samples had been measured in 2021 and were stored until now at 25 °C under air. The results are provided in Figure S22. Thereby, we also assessed the response of these analytical method varying in signal generation principle to aging induced changes of the particle surface and the silica network. With the optical assay, we could detect less surface amino FGs compared to the newer batches after this long storage period. However, with qNMR, measuring solutions of dissolved aminated SiO_2 NPs, amino FGs were

still detectable, although their number was reduced to 39% and 51%, respectively compared to the data found in the first ILC of NC-20 as to be expected. The larger NC-50 and NC-120 showed smaller decrease of accessible amino FGs in the fluram assay. Interestingly, the potentiometric titration, measuring dispersed particles, provided an amount of amino FGs of 215 ± 10 µmol/g for NC-120 that closely matched with the qNMR results. Apparently, protons can penetrate the silica network and still access amino FGs buried within the silica network, while larger dye reporters can only react and thereby detect surface amino FGs.

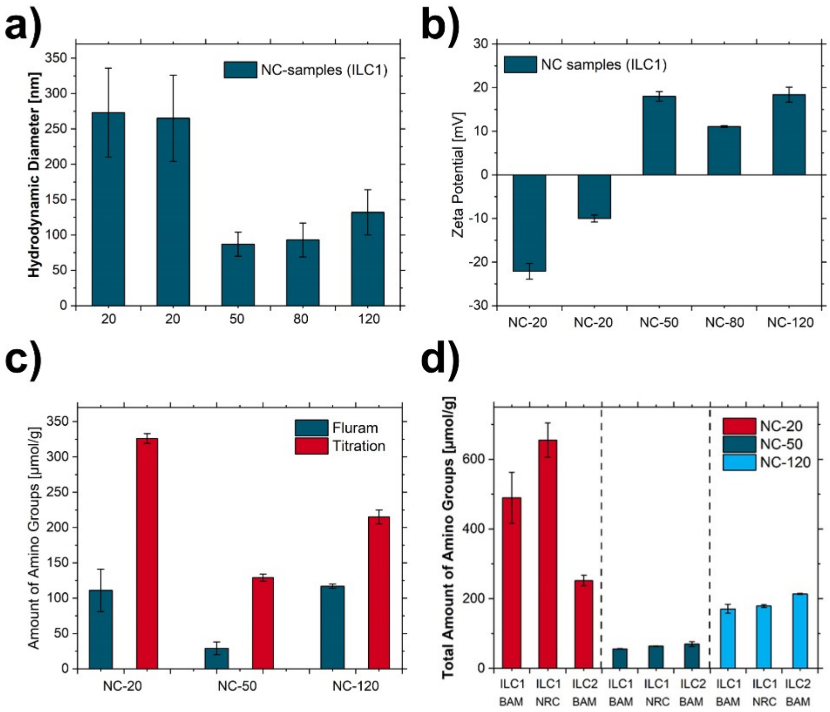

Figure S25. Overview of the multimethod approach (a) DLS, b) Zeta, c) screening with fluram assay and titration, d) qNMR) for NC-20 (JRC0486), NC-50 (MEL0032), NC-80 and NC-120 (JEA0209) measured in the first and second ILC, indicating the stability of the particles over time.

8. References

(1) Tavernaro, I.; Cavelius, C.; Peuschel, H.; Kraegeloh, A. Bright fluorescent silica-nanoparticle probes for high-resolution STED and confocal microscopy. *Beilstein J Nanotechnol* **2017**, *8*, 1283-1296. DOI: 10.3762/bjnano.8.130.

(2) Schmidt, S.; Tavernaro, I.; Cavelius, C.; Weber, E.; Kumper, A.; Schmitz, C.; Fleddermann, J.; Kraegeloh, A. Silica Nanoparticles for Intracellular Protein Delivery: a Novel Synthesis Approach Using Green Fluorescent Protein. *Nanoscale Res Lett* **2017**, *12* (1), 545. DOI: 10.1186/s11671-017-2280-9.

(3) Stöber, W.; Fink, A.; Bohn, E. Controlled Growth of Monodisperse Silica Spheres in the Micron Size Range. *Journal of Colloid and Interface Science* **1968**, *26*, 62-69. DOI: https://doi.org/10.1016/0021-9797(68)90272-5.

(4) Tavernaro, I.; Matiushkina, A.; Rother, K. S.; Mating, C.; Resch-Genger, U. Exploring the potential of simple automation concepts for quantifying functional groups on nanomaterials with optical assays. *Nano Research* **2024**, *17* (11), 10119-10126. DOI: 10.1007/s12274-024-6970-1.

(5) ASTM. *ASTM E2834-12 Standard Guide for Measurement of Particle Size Distribution of Nanomaterials in Suspension by Nanoparticle Tracking Analysis (NTA)*; 2012. DOI: 10.1520/E2834-12. *International Organization for Standardization ISO*

19430:2016, Particle size analysis-Particle tracking analysis (PTA) method.; 2016. DOI: https://www.iso.org/standard/64890.html.

(6) Dietrich, P. M.; Streeck, C.; Glamsch, S.; Ehlert, C.; Lippitz, A.; Nutsch, A.; Kulak, N.; Beckhoff, B.; Unger, W. E. Quantification of silane molecules on oxidized silicon: are there options for a traceable and absolute determination? *Anal Chem* **2015**, *87* (19), 10117-10124. DOI: 10.1021/acs.analchem.5b02846.

(7) Kunc, F.; Nirmalananthan-Budau, N.; Ruhle, B.; Sun, Y.; Johnston, L. J.; Resch-Genger, U. Interlaboratory Comparison on the Quantification of Total and Accessible Amine Groups on Silica Nanoparticles with qNMR and Optical Assays. *Anal Chem* **2021**, *93* (46), 15271-15278. DOI: 10.1021/acs.analchem.1c02162.

(8) Kunc, F.; Balhara, V.; Brinkmann, A.; Sun, Y.; Leek, D. M.; Johnston, L. J. Quantification and stability determination of surface amine groups on silica nanoparticles using solution NMR. *Anal Chem* **2018**, *90* (22), 13322-13330. DOI: 10.1021/acs.analchem.8b02803.

(9) C. I. Crucho, C. Baleizao and J. P. Farinha, *Anal Chem*, 2017, **89**, 681-687.
https://doi.org/10.1021/acsbiomaterials.1c00440

(10) I.-L Jung H.-S. Jung, D.-S. Moon, J.-K. Lee, *J. Nanomaterials*, 2012, 593471. **https://doi.org/10.1155/2012/593471**

11) Geissler, D.; Nirmalananthan-Budau, N.; Scholtz, L.; Tavernaro, I.; Resch-Genger, U. Analyzing the surface of functional nanomaterials-how to quantify the total and derivatizable number of functional groups and ligands. *Mikrochim Acta* **2021**, *188* (10), 321. DOI: 10.1007/s00604-021-04960-5. Krishna, D. N. G.; Philip, J. Review on surface-characterization applications of X-ray photoelectron spectroscopy (XPS): Recent developments and challenges. *Applied Surface Science Advances* **2022**, *12*. DOI: 10.1016/j.apsadv.2022.100332.

(12) Shinotsuka, H.; Nagata, K.; Siriwardana, M.; Yoshikawa, H.; Shouno, H.; Okada, M. Sample structure prediction from measured XPS data using Bayesian estimation and SESSA simulator. *Journal of Electron Spectroscopy and Related Phenomena* **2023**, *267*. DOI: 10.1016/j.elspec.2023.147370.

(13) Bennet, F.; Müller, A.; Radnik, J.; Hachenberger, Y.; Jungnickel, H.; Laux, P.; Luch, A.; Tentschert, J. Preparation of Nanoparticles for ToF-SIMS and XPS Analysis. *J. Vis. Exp.* **2020**, *163*, e61758. DOI: doi:10.3791/61758.
